# Trauma-specific mindfulness-based cognitive therapy for women with post-traumatic stress disorder and a history of domestic abuse: intervention refinement and a randomised feasibility trial (coMforT study)

**DOI:** 10.1186/s40814-023-01335-w

**Published:** 2023-07-03

**Authors:** Natalia V. Lewis, Alison Gregory, Gene S. Feder, Aishlyn Angill-Williams, Sophie Bates, Joel Glynn, Gemma Halliwell, Claire Hawcroft, David Kessler, Michael Lawton, Rwth Leach, Sarah Millband, Katherine Pitt, Stan Zammit, Alice Malpass

**Affiliations:** 1 NIHR Bristol Biomedical Research Centre, University Hospitals Bristol and Weston NHS Foundation Trust and University of Bristol, Canynge Hall, Bristol, BS8 2PS UK; 2 Bristol Medical School (PHS), University of Bristol, Canynge Hall, Bristol, BS8 2PS UK; 3 NIHR Clinical Research Network (CRN), Bristol Medical School (PHS), University of Bristol, Oakfield House, Bristol, BS8 2BN UK; 4 Division of Psychological Medicine and Clinical Neurosciences, Cardiff University, Cardiff, CF24 4HQ UK

**Keywords:** Mindfulness, Mind-Body therapies, Mindfulness-based cognitive therapy, Psychosocial intervention, Domestic violence, Intimate partner biolence, Stress disorders, post-traumatic, Feasibility studies, pilot projects

## Abstract

**Background:**

Women who have experienced domestic violence and abuse (DVA) are at increased risk of developing post-traumatic stress disorder (PTSD) and complex PTSD (CPTSD). In 2014–2015, we developed a prototype trauma-specific mindfulness-based cognitive therapy curriculum (TS-MBCT) for the treatment of PTSD in a DVA population. This study aimed to refine the prototype TS-MBCT and evaluate the feasibility of conducting a randomised controlled trial (RCT) testing its effectiveness and cost-effectiveness.

**Methods:**

Intervention refinement phase was informed by evidence synthesis from a literature review, qualitative interviews with professionals and DVA survivors, and a consensus exercise with experts in trauma and mindfulness. We tested the refined TS-MBCT intervention in an individually randomised parallel group feasibility trial with pre-specified progression criteria, a traffic light system, and embedded process and health economics evaluations.

**Results:**

The TS-MBCT intervention consisted of eight group sessions and home practice. We screened 109 women in a DVA agency and recruited 20 (15 TS-MBCT, 5 self-referral to National Health Service (NHS) psychological treatment), with 80% follow-up at 6 months. Our TS-MBCT intervention had 73% uptake, 100% retention, and high acceptability. Participants suggested recruitment via multiple agencies, and additional safety measures. Randomisation into the NHS control arm did not work due to long waiting lists and previous negative experiences. Three self-administered PTSD/CPTSD questionnaires produced differing outcomes thus a clinician administered measure might work better. We met six out of nine feasibility progression criteria at green and three at amber targets demonstrating that it is possible to conduct a full-size RCT of the TS-MBCT intervention after making minor amendments to recruitment and randomisation procedures, the control intervention, primary outcomes measures, and intervention content. At 6 months, none of the PTSD/CPTSD outcomes ruled out a clinically important difference between trial arms indicating that it is reasonable to proceed to a full-size RCT to estimate these outcomes with greater precision.

**Conclusions:**

A future RCT of the coMforT TS-MBCT intervention should have an internal pilot, recruit from multiple DVA agencies, NHS and non-NHS settings, have an active control psychological treatment, use robust randomisation and safety procedures, and clinician-administered measures for PTSD/CPTSD.

**Trial registration:**

ISRCTN64458065 11/01/2019.

**Supplementary Information:**

The online version contains supplementary material available at 10.1186/s40814-023-01335-w.

## Key messages regarding feasibility

- Our proof-of-concept research identified areas of uncertainty regarding a full-scale randomised controlled trial (RCT), including the choice of our control intervention, recruitment of women with post-traumatic stress disorder/complex post-traumatic stress disorder (PTSD/CPTSD) and a history of domestic violence and abuse (DVA), randomisation into intervention and control arms, retention in the trial, uptake and engagement with trauma-specific mindfulness-based cognitive therapy (TS-MBCT), and data collection methods.
- It is possible to conduct a full-size RCT of the TS-MBCT intervention after making minor amendments to recruitment and randomisation procedures, the control intervention, primary outcomes measures, and intervention content.
- A future RCT of the coMforT TS-MBCT intervention should have an internal pilot, recruit from multiple DVA agencies, NHS and non-NHS settings, have an active control psychological treatment, use robust randomisation and safety procedures, and clinician-administered measures for PTSD/CPTSD.

## Background

Domestic violence and abuse (DVA) is a highly prevalent public health and clinical problem associated with increased morbidity [1], mortality [2], and use of healthcare services [3]. DVA encompasses acts of physical aggression, sexual abuse and coercion, psychological abuse, and controlling behaviours by a current or former intimate partner, or an adult family member, resulting in physical, sexual, or psychological harm [4]. DVA is recognised as a complex trauma because of the chronicity and complexity of the violence and abuse and the impact on affect regulation, changes in consciousness, sense of self, relationships, and belief systems [5]. Although DVA is not confined to acts perpetrated by men against women, or to heterosexual relationships, the associated morbidity and mortality are highest among women, with the greatest damage to their mental health [6]. Systematic reviews reported comorbid post-traumatic stress disorder (PTSD), depression, anxiety, and substance abuse as the most common mental health sequelae [7, 8]. The odds ratio (OR) for lifetime partner violence among women with PTSD was 7.34 (95% confidence interval (CI) 4.50 to 11.98) [8]. Children of mothers who have experienced DVA and PTSD are at increased risk of behavioural and health problems [9]. Women exposed to DVA are at higher risk of developing complex PTSD (CPTSD) [10]. Research suggests that CPTSD is twice as prevalent as PTSD among women survivors of intimate partner violence (39.50% vs 17.90%) [11]. Unlike standard PTSD, the definition, classification, and evidence-based treatment for CPTSD are in development [12]. The UK Psychological Trauma Society (UKSPTS) recommends a phased-based approach to treatment of CPTSD and highlights insufficient evidence to recommend any particular therapy over another [13].

Clinical guidelines recommend individual trauma-focused cognitive behavioural therapy (TF-CBT) or eye movement desensitisation and reprocessing (EMDR) for women with PTSD who are no longer experiencing DVA, with the caveats that these interventions are delivered by professionals with an understanding of DVA [14, 15]. However, a recent systematic review found that trauma-focused therapies are associated with greater dropout compared to those without a trauma focus [16]. Women who have experienced DVA have reported that standard trauma-focused treatments might not be acceptable because of the barriers to access (e.g. childcare, work), and the potential for re-traumatisation from revisiting traumatic experiences [17]. Another systematic review concluded that psychosocial interventions for women with a history of DVA had the greatest impact when they took a holistic approach and provided individualised and trauma-informed support [18].

In contrast to recommended trauma-focused methods, mindfulness-based interventions for PTSD use an acceptance mode for responding to distressing experiences. By not including exposure work and using a holistic mind-body approach, these interventions may be more acceptable to survivors of DVA. The UKPTS guideline on CPTSD recommends mindfulness as a component in skills-training packages during phase 1: stabilisation and psychoeducation [13]. Recent reviews have identified the need for effective trauma-specific mindfulness interventions for the treatment of PTSD/CPTSD, evaluated by randomised controlled trials (RCTs) and with special attention to adverse events [19–21].

The coMforT (Mindfulness for Trauma) study is built on our proof-of-concept research. In 2014–2015, we conducted a literature review of mindfulness-based interventions and consulted patient and professional stakeholders, aiming to identify potential adaptations to standard mindfulness treatment in order to address the specific vulnerabilities of DVA survivors. Informed by this work, a mindfulness teacher, with lived experience of DVA and specialism in trauma, developed an initial prototype trauma-specific MBCT curriculum (TS-MBCT-1) (Millband S: How can an adapted MBCT course meet the specific vulnerabilities of women survivors of domestic violence and abuse?, unpublished thesis), by adapting the standard mindfulness-based cognitive therapy (MBCT) programme for depression prevention [22]. We plan to conduct a randomised controlled trial (RCT) of the TS-MBCT intervention. Our proof-of-concept research identified areas of uncertainty regarding a proposed trial, including the choice of our control intervention, recruitment of women with PTSD/CPTSD from a DVA population, randomisation into intervention and control arms, retention in the trial, uptake and engagement with TS-MBCT, and data collection methods. The coMforT study aimed to develop an evidence-based, acceptable, and feasible TS-MBCT intervention, and to evaluate the feasibility of conducting an RCT testing the effectiveness and cost-effectiveness of the developed intervention in reducing PTSD/CPTSD symptoms in a DVA population.

## Methods

**Fig. 1:**
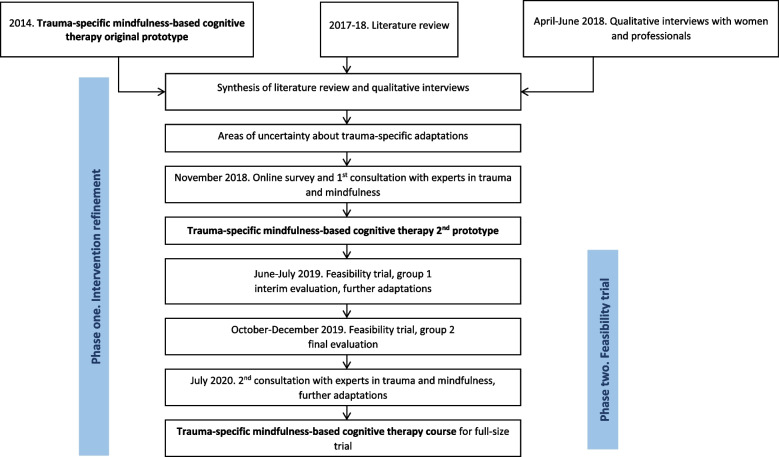
coMforT study design

Informed by guidance on the development of complex healthcare interventions [23] and feasibility studies [24], we conducted coMforT in two overlapping phases (Fig. 1).

We published a detailed protocol elsewhere [25] and registered the feasibility trial [26]. An advisory group of women with lived experience of DVA advised the study team. A steering committee of independent experts oversaw the conduct of the study and advised on progression to a full-size RCT.

### Qualitative methods

We conducted qualitative semi-structured interviews in both phases. Interviews were audio-recorded, transcribed verbatim, anonymised, and uploaded into NVivo 12 software. We used inductive and deductive coding, applying constant comparison techniques [27, 28], to develop themes about trauma-specific adaptations to the curriculum (intervention refinement phase), and about the acceptability of the intervention and trial procedures (feasibility trial). We applied a framework method [29]. Our initial analytical coding frame was based on the framework for modifications and adaptations of evidence-based interventions [30], and the framework of mindfulness-based programmes fidelity [31]. We organised codes under two analytical themes of trauma-specific adaptations to the curriculum content and course context. We applied the initial coding frame across all qualitative datasets and refined it as the analysis progressed.

### Phase 1: intervention refinement

The aim of phase 1 was to develop a TS-MBCT intervention that is acceptable to the DVA population and feasible to deliver in an RCT. The intervention refinement work involved evidence synthesis from an updated 2014 literature review, qualitative interviews with women and professionals, and a 2-stage consensus exercise with experts in trauma and mindfulness (Fig. 1, Table 1).

**Table 1:** Phase 1: intervention refinement. Study assessments

Procedures	Timepoint	Measurement tool
Socio-demographic data-women	At the start of the qualitative interviews	Bespoke questionnaire (age, ethnicity, education, employment, relationship status, time since end of abusive relationship, previous talking therapy)
Face-to-face/phone semi-structured qualitative interviews with women	Before the first mindfulness group	Topic guide exploring experiences of recovery after DVA, and any psychological support received (Supplementary file 1)
Socio-demographic data-professionals	At the start of the qualitative interviews	Bespoke questionnaire (age, gender, job title, years of delivering psychological therapy to people affected by violence and trauma)
Face-to-face/phone/Skype semi-structured qualitative interviews with professionals	Before the first mindfulness group	Topic guide exploring experiences of providing psychological interventions to people affected by violence and trauma (Supplementary file 1)
Online survey with experts in trauma and mindfulness	Before the first mindfulness group	Bespoke questionnaire: years of practice, 15 statements about trauma-specific modifications to a standard mindfulness-based cognitive therapy course grouped under three categories: participants, curriculum, and teacher. 5-point Likert scale (strongly disagree to strongly agree), free text comments (Supplementary file 2)
Remote consultations with experts in trauma and mindfulness	1. Before feasibility trial 2. Six months post-randomisation	1. Agenda covering seven statements from the survey that produced contention 2. Agenda covering areas of uncertainty identified through the process evaluation of the trial mindfulness groups

#### Literature review

We searched MEDLINE for studies of any design that evaluated mindfulness-based programmes for people affected by childhood abuse or DVA. Results and discussion sections of included papers were treated as primary qualitative data with deductive coding of text which reported trauma-specific adaptations to standard mindfulness interventions.

#### Qualitative semi-structured interviews with women and professionals

Interviews explored participants’ views on proposed TS-MBCT-1. Recruitment via email to patient and professional networks drew a purposive sample of (i) women aged 18+ years who self-identified as having experienced DVA and mental health problems, and (ii) professionals working with people affected by trauma. Researchers conducted interviews at university offices or online. Topic guides included a vignette about the TS-MBCT-1 prototype and questions about intervention feasibility, acceptability, barriers, and facilitators to women, group logistics, and trial procedures (Supplementary file 1).

#### Two-stage consensus exercise with experts in trauma and mindfulness

Our literature review and qualitative interviews identified conflicting perspectives on proposed adaptations and raised new questions about the inclusion/exclusion of participants, the content and format of the TS-MBCT-1 prototype, and the teachers’ training and qualifications. The aim of the consensus exercise was to develop pragmatic solutions for further intervention refinement and trial procedures. We sent an expression-of-interest email with an online survey to networks of mindfulness teachers inviting those who had taught mindfulness-based programmes to people affected by trauma, including DVA. The anonymous survey included 15 statements relating to the remaining uncertainties (Supplementary file 2). We imported the responses into Microsoft Excel and analysed quantitative data with descriptive statistics and narrative text. We manually coded free text comments on the framework themes of trauma-specific adaptations to curriculum content and context. We then invited survey respondents to two virtual consultations with the study researchers and the mindfulness teacher. In the first consultation, attendees formulated pragmatic resolutions of conflicting statements. In the second, carried out post-trial, the respondents agreed further refinements to the intervention. Discussion about remaining areas of uncertainty and agreed solutions were summarised narratively.

#### Evidence synthesis

We integrated the findings from the literature review, qualitative interviews, and consensus exercise into an evidence matrix. We categorised evidence from the three sources as no evidence, limited evidence (single study), agreement (different studies/contributors reported converging perspectives), and disagreement (different studies/contributors reported diverging or conflicting perspectives). We further scrutinised gaps in the evidence, limited evidence, and disagreements until consensus was reached in the form of agreed terms and conditions.

### Phase 2: feasibility trial

We conducted an individually randomised parallel group feasibility trial with pre-specified progression criteria and a traffic light system [32], and an embedded mixed-method process evaluation and health economics evaluation. Women were screened, assessed at baseline, randomised 2:1 into intervention (TS-MBCT group) and control (self-referral to psychological therapy in the National Health Service (NHS)), and followed-up 6 months post-randomisation. We ran two consecutive TS-MBCT groups, with findings from the interim process evaluation informing refinements of the TS-MBCT curriculum and trial procedures between the two groups (Fig. 1).

#### Data collection

We used convenience sampling to recruit trial participants during 2-month periods (plus 1 buffer month) prior to pre-scheduled TS-MBCT group dates.

#### Initial screening

The recruitment sites were four charitable-sector specialist DVA agencies in Southwest England, UK. The study researchers trained agency support workers on the recruitment protocol, and regularly visited the sites. To ensure that potential participants received specialist DVA support, agency workers were asked to carry out an initial screening of all female clients near to case closure, or on a waiting list for any of the agency services (Table 2).

**Table 2:** Phase 2. Feasibility trial with embedded process evaluation and economic evaluation. Study assessments

Procedures	Initial screening by recruitment site worker	Final screening by study researcher	Baseline assessment by study researcher	6-month follow up by study researcher	Measurement tool
Eligibility assessment					
Age	x		x		Bespoke socio-demographic questionnaire: age, ethnicity, education, employment, marital status, children, housing, and financial situation
Speaking and understanding English	x				Recruitment site worker’s judgement
Diagnosed psychosis, bipolar disorder, personality disorder	x				Recruitment site case record
Current psychological therapy	x				Recruitment site case record
Readiness to start mindfulness group or alternative talking therapy on the NHS	x				Woman’s own judgement
Post-traumatic stress disorder (PTSD)		x			The Primary Care PTSD Screen for DSM-5 (PC-PTSD-5) [33]
Current alcohol dependence		x			The Alcohol Use Disorders Identification Test Consumption (AUDIT-C) [34]
Current drug dependence		x			The Drug use Disorders Identification Test (DUDIT) [35]
Suicide history		x			Bespoke questions: • I made plans to end my life in the last 2 weeks • I made attempts to end my life in the last 12 months
Trial assessments					
Live events			x		The Life Events Checklist for DSM-5 (LEC-5) Standard [36]
Clinically important symptoms of PTSD			x	x	The PTSD Checklist for DSM-V (PCL-5) [36]
Clinically important symptoms of complex PTSD			x	x	The International Trauma Questionnaire (ITQ) [37]
Depression			x	x	The Patient Health Questionnaire-9 (PHQ-9) [38]
Anxiety			x	x	Generalized Anxiety Disorder-7 (GAD-7) [39]
Adverse childhood experiences			x		Brief screening version of the Childhood Trauma Questionnaire [40]
Domestic violence and abuse			x	x	Composite Abuse Scale Revised-Short Version [41]
Dissociative symptoms			x	x	The Severity of Dissociative Symptoms—Adult (Brief Dissociative Experiences Scale [DES-B]—Modified) [42]
Self-compassion			x	x	Self-Compassion Scale–Short Form (SCS-SF) [43]
Embedded health economic evaluation					
Health related quality of life, women			x	x	EQ-5D-5L [44]
Health-related quality of life, index child			x	x	KIDSCREEN-10 Index. Health Questionnaire for Children and Young People. Parent Version [45]
Resource use				x	Bespoke
Intervention cost					Study documentation
Embedded process evaluation					
Qualitative semi-structured interviews with trial participants				x	Topic guide about experiences of the trial and interventions (Supplementary file 3)
Feedback form (intervention arm)				x	Bespoke

Initial eligibility criteria were: female, aged 18+ years old, able to speak and understand English, no diagnosis of psychosis or bipolar or personality disorder, no current psychological therapy, feeling ready to attend a mindfulness group or an alternative NHS psychological therapy. The agency workers emailed the research team with contact and safety information of clients who met the initial eligibility criteria, were interested in the study, and agreed to the sharing of their details.

#### Final screening

The study researcher made four attempts to contact each potential participant and arrange face-to-face screening at a safe and convenient location. Women completed four questionnaires. At the final screening stage, we included women who self-reported clinically important symptoms of PTSD on the Primary Care PTSD Screen for DSM-5 (PC-PTSD-5) [33]. They did not have to have a formal diagnosis of PTSD established by a clinician [46]. We excluded women who had current alcohol dependency on the Alcohol Use Disorders Identification Test (AUDIT) [34], drug dependency on the Drug Use Disorders Identification Test (DUDIT) [35] or who had made suicide plans in the last 2 weeks or suicide attempts in the last 12 months.

#### Baseline assessment

Women completed baseline questionnaires (Table 2). If the woman had children, the researcher used a bespoke Access database to randomly select an index child for inclusion in the study.

#### Randomisation

The recruiting researcher phoned/emailed the remote randomisation service. The Database Manager used blocked randomisation to form the allocation list for the two trial arms. A computer random number generator was used to select random permuted blocks with a block size of 18 and a 2:1 ratio. The researcher notified women of their allocation and conveyed details of those randomised into the intervention arm to the mindfulness teacher. Due to the nature of the psychological interventions in both arms, it was not possible to blind participants, intervention providers, or researchers to the group allocation.

#### Follow-up 6-month post-randomisation

A study researcher maintained contact with participants via monthly check-in texts, emails, and phone calls. Participants received a £20 shopping voucher and reimbursement of travel and childcare expenses for attending each assessment and intervention session. At 6 months post-randomisation, the researcher arranged a face-to-face assessment, at the woman’s choice of location. Women completed follow-up questionnaires. Due to COVID-19 lockdown, one participant received and returned the questionnaire via post.

#### Process evaluation

We collected process data after the first TS-MBCT group in the intervention arm, and at the 6-month follow-up in both arms. Women in the intervention arm completed a Home Practice Feedback Form after each session and an Evaluation Form at the end of the course. During the monthly check-in contacts, a study researcher obtained women’s permission to be contacted for a qualitative interview, and conveyed these details to two qualitative researchers who arranged face-to-face interviews in university offices or the woman’s home. The feedback forms and interviews (Supplementary file 3) explored women’s experiences of the interventions and trial procedures.

#### Health economics evaluation

We recorded information about the resources used in the delivery of the TS-MBCT intervention. At 6-month follow-up, women completed a bespoke resource use questionnaire capturing theirs, and an index child’s, use of health and social care services over the study period. Quality of life was measured with the EQ-5D-5L (women) [44] and KIDSCREEN (an index child) [45] questionnaires during the baseline and 6-month follow-up assessments.

### Outcomes and measures

#### Feasibility outcomes

We calculated the rates of recruitment (primary feasibility objective), intervention uptake, retention, and follow-up, and qualitatively assessed the acceptability of the intervention and trial design. We extracted data from study logs, mindfulness teacher records, TS-MBCT Feedback and Evaluation Forms, and administrative NHS data.

#### Candidate clinical outcomes in a full trial

We tested standardised validated questionnaires for measuring clinically important symptoms of PTSD and CPTSD (candidate primary outcomes), dissociative symptoms, depression, anxiety, DVA re-experience, and self-compassion (candidate secondary outcomes).

#### Adverse events

During the monthly checks, 6-month follow-up, and process evaluation interviews, study researchers asked women about any adverse events. The TS-MBCT teacher was available at the beginning and end of sessions, and between sessions, if women felt that their symptoms were worsening and wanted to talk. Adverse events were processed according to the trial protocol [25].

#### Analysis

We entered questionnaire responses into REDCap software, and prepared and analysed quantitative data in STATA16. To give an acceptable precision for estimating the proportion of eligible women consenting to participate, we aimed to screen 120 women and to recruit 36 (recruitment ratio: 30% of those eligible). Assuming a 33% drop out rate before the start of the intervention, we inflated the sample size to 54 [25]. Following recommendations on developing progression criteria for pilot studies [32], we prespecified progression criteria and a traffic light system for decision-making about progression to a full trial. All green targets met: progress to the full trial. Green and/or amber targets met: Study Steering Group discuss problems with study management group, and progress if strategies are available to improve the intervention and trial protocol. Red targets met: consult Steering Committee and funder if progression is justified [25]. We reported feasibility outcomes by mapping on the progressions criteria and traffic lights. For criteria that were based on quantitative and qualitative data, we integrated findings through joint display of statistics-by-themes [47]. Since this was a feasibility study, we did not perform significance testing for effect sizes in candidate clinical outcomes for a full trial, instead calculating descriptive statistics with 95% CI. We reported differences in means and 95% CIs between the two arms at 6 months.

## Results

### Phase 1: intervention refinement

#### Literature review

We found a gap in the UK evidence, and a lack of detail on trauma-specific adaptations to standard mindfulness interventions reported in eight studies which addressed some areas of uncertainty relevant to the design of our trial [19, 48–54].

#### Qualitative interviews with women and professionals

Between April and June 2018, 20 women expressed an interest, 3 were ineligible and 10 did not respond. Of 40 professionals who expressed interest, 2 were ineligible, and 16 did not respond. The researchers interviewed 7 women (6 face-to-face, 1 telephone) and 13 professionals (7 face-to-face, 4 telephone, 2 Skype). Women’s ages ranged from 25 to 64 years; 3 were White British, 2 White European, and 2 British Asian; 5 worked; and 6 had children. Eleven of the 13 professionals were female; 7 worked in the NHS, 4 in charitable sector, and 2 were self-employed; experience of trauma work ranged from 1 to 30 years.

#### Two-stage consensus exercise with experts in trauma and mindfulness

In November 2018, 11 experts expressed an interest in the online survey, and 8 provided responses. Their experience of working with people affected by trauma ranged from 1 to 23 years. Two experts from the USA and one from the UK also attended the first consultation in November 2018, and the same two USA experts attended the second consultation in July 2020.

#### Evidence synthesis

Whilst integrating evidence from the literature and interviews using the matrix, we identified conflicting perspectives within the sub-themes of (i) the curriculum content (the direction of change in psychological processes), and (ii) the context (the exclusion of women with poor English, substance dependence, suicidal behaviour, concurrent psychological therapy; TS-MBCT as a first-line therapy; qualifications and experience of TS-MBCT teachers; power imbalance in the teacher-participant relationship). The conflicting perspectives were scrutinised during the consultation with experts in trauma and mindfulness. Following nuanced and insightful discussions, the experts and coMforT researchers agreed the pragmatic terms and conditions for resolving these ambiguities (Supplementary file 4). The mindfulness teacher used the matrix to produce a second prototype TS-MBCT (TS-MBCT-2) for the feasibility trial. Researchers used this matrix to refine the trial procedures.

#### Interventions

##### TS-MBCT

We adapted the standard MBCT curriculum [22] to address the vulnerabilities of women affected by DVA, which include trauma-related patterns of avoidance, re-experiencing, and reactivity [55]. We retained the structure of a standard 8-week MBCT course, adapted core themes and specific curriculum elements, including mindfulness practices, and added new psycho-educational material. In making and reporting adaptations, we considered the essential and flexible ingredients of mindfulness-based programmes, as recommended by Crane et al. [56] The adaptations were informed by trauma theory [5], the framework of mindfulness-based programme fidelity [56] and our evidence synthesis. Adaptations were made to embed safety in all aspects of the course from the orientation and assessment procedures and throughout the course curriculum itself. The course was delivered in 2-h sessions over 8 weeks. Risks in relation to travel and geographic location, as well as consideration of each woman’s readiness to participate and safety in her own home, were assessed in individual O&A meetings. Mindfulness practice guidance was adapted to include a stronger emphasis on choice. A menu of practices of varying lengths were offered during sessions and to support home practice. Adaptations were made to the amount of home practice required and psycho-education was included about the psychological distress associated with trauma and the ways in which mindfulness could support women with recognising and responding wisely to patterns of avoidance, reactivity, and re-experiencing. Movement practices to support self-regulation were included in each session and repeated emphasis that the attitude of acceptance that underpins mindfulness-based programmes relates to cultivating acceptance of internal experiences and not to harmful behaviour from others was offered. Ways of working with difficulty, including flashbacks and overwhelm, were included from the first session of the course. Adaptations to experiential learning elements of the curriculum included mindful communication exercises to address DVA as a relational trauma that impacts connection with self and others (Table 3).

**Table 3:** Overview of the trauma-specific mindfulness-based cognitive therapy reported in the Template for Intervention Description and Replication (TIDieR) checklist [57]

TIDieR item	Trauma-specific mindfulness-based cognitive therapy description
1. Brief name	Trauma-specific mindfulness-based cognitive therapy (TS-MBCT)
2. Why	The mindfulness-based cognitive therapy (MBCT) curriculum for depression relapse [22] adapted to address specific vulnerabilities of women affected by domestic violence and abuse. Adaptations were informed by trauma theory [5], the framework of mindfulness-based programmes (MBPs) fidelity [56] and our evidence synthesis.
3. What materials	Trauma-specific adaptations to the standard MBCT curriculum mapped on the coding system for modifications and adaptations of evidence-based interventions [30]. Tailoring/Tweaking/Refining: - Refinement of assessment and orientation process, with a stronger focus on safety. - Tailoring practice guidance to emphasise choice and support the process of recognising, responding to and working wisely with overwhelming experience and providing trauma-sensitive audio recordings for home practice - Tweaking language and vocabulary on handouts. Adding elements: - Psycho-education on developing awareness of responses to overwhelm and inclusion of the Zones of Proximal Development Model [58] - Movement practice for responding to overwhelm. - Psycho-education on how the tone of negative inner dialogue relates to the experience of domestic violence and abuse. - Psycho-education on the relationship between aversion and trauma and how patterns of reactivity maintain distress. - Psycho- education on PTSD and inclusion of the Window of Tolerance Model [22] - Difficult Communications Calendar (from Mindfulness-Based Stress Reduction). - Sea of Reactions experiential exercise (from MBCT-Ca). - Additional handouts to support learning and home practice. Removing elements: - Yoga–replaced with Qi Gong. Shortening/condensing: - Shorter practices. Lengthening/extending: - Offering a menu of home practices to choose from - Attentional focus practice extended to include ‘impulses’, in addition to body sensations, thoughts and feelings. - Extending the theme of acceptance to include acceptance of all internal experience, including flashbacks Re-ordering elements: - Awareness of patterns of aversion, reactivity and re-experiencing included from week 1, rather than week 4. Repeating elements: - Stronger and repeated emphasis on permission and making wise choices. - Repeated emphasis that the attitude of acceptance in MBPs relates to acceptance of internal experiences and not to harmful behaviour from others
4. What procedures	Adaptations to MBCT procedures all relate to enhanced safety measures—both before and during the course. In response to women’s suggestions, we developed coMforT app for supporting home practice. Following advice from the study advisory group and professional stakeholders, we offered women a £20 shopping voucher and reimbursement of transport expenses and childcare for attending each session.
5. Who provided	Female mindfulness teacher (author SM) and teacher assistant. SM has a Masters in Teaching mindfulness from Bangor University and a Certificate of Competence to Teach Mindfulness—she was assessed as proficient, following the completion of 5 years of training in 2013. SM is listed with BAMBA and meets the GPG for MBP teachers in the UK, which include developing and maintaining a personal mindfulness practice, attending annual retreats and completing required continuing professional development. A master’s in psychology graduate assisted SM at group 1. A member of the coMforT advisory group assisted at group 2.
6. How	In person teacher-led delivery to a group of up to eight.
7. Where	Room in a community centre located in an urban area of high deprivation
8. When and how much	2-h session was delivered weekly over 8 weeks, though adaptations were made to how much home practice was expected—participants were offered a menu of practices of different lengths and encouraged to make choices about the amount of weekly home practice undertaken in response to their individual vulnerabilities.
9. Tailoring	Individual needs and vulnerabilities were met by offering individual meetings with the teacher, as well as phone and email support in between session where appropriate and with sensitivity to safety
10. Modifications	Modifications to practice guidance were made between groups 1 and 2 to deepen trauma-sensitivity in the language used.
11. How well planned	Recording sessions was not appropriate for this population. We co-developed an MBP fidelity tool with input from a member of the coMforT advisory group. She and a qualitative researcher attended the TS-MBCT group, completed the checklist for each session and collated the results.
12. How well actual	Due to funding restrictions, we did not access fidelity of the course in the feasibility trial. We plan to use the MBP fidelity tool in the full-size trial.

##### Control intervention

Women randomised to the control arm were able to self-refer to the local NHS psychological support service, which provides stepped psychological treatment for mild to moderate mental health problems (step 2), PTSD (step 3), and CPTSD (step 4).

### Phase 2: feasibility trial

The MBCT-trained teacher, with specialism in trauma, and a teaching assistant, ran two TS-MBCT groups (3 June–29 July 2019 and 14 October–8 December 2019), with seven women allocated to group 1 and eight to group 2. Six out of nine progression criteria (acceptability of recruitment, follow-up procedures, data collection methods; follow-up rate; uptake of and retention in TS-MBCT group) were met at green targets, and three (recruitment rate; acceptability of randomisation and TS-MBCT intervention) at amber (Table 4). The Steering Committee gave the amber light to proceed to a full-size RCT after making amendments to the recruitment and randomisation procedures, and to the intervention content.

**Table 4:** Pre-specified progression criteria with a traffic light system for decision making about proceeding to a full-size trial

*N*	Criteria	Measurement	Traffic light system for proceeding to a full-size trial					
			Green light: go—proceed		Amber light: amend—proceed with caution		Red light: stop—do not proceed	
			Planned	Achieved	Planned	Achieved	Planned	Achieved
1	Recruitment	Number of participants recruited over a 6-month period	≥ 24	–	23–12	12	< 12	–
2	Acceptability of recruitment procedures	Qualitative process evaluation	Most participants find acceptable or only minor amendments needed	11 interviewees found procedures acceptable and suggested additional recruitment pathways.	Conflicting views or larger changes needed	–	Most participants find unacceptable	–
3	Acceptability of randomisation procedure	Qualitative process evaluation	Most participants find acceptable or only minor amendments needed	**—**	Conflicting views or larger changes needed	One interviewee suggested to clarify the procedure. Some participants felt that randomisation into NHS psychological support service was not appealing.	Most participants find unacceptable	–
4	Follow-up (total and by trial arms)	Proportion of enrolled participants who provided primary outcome data at 6 months post-randomisation (out of those randomised)	> 50%	16/20 women (80%): intervention arm 13/15 (87%) control arm 3/5 (60%)	31–50%	–	< 50%	–
5	Acceptability of follow-up procedure (total and by trial arms)	Qualitative process evaluation	Most participants find acceptable or only minor amendments needed	For some women the questions asked at follow-up could be triggering.	Conflicting views or larger changes needed	–	Most participants find unacceptable	–
6	Uptake of trauma-specific mindfulness-based cognitive therapy (intervention arm)	Proportion of participants who started the mindfulness group (out of those randomised to the intervention arm)	≥ 70%	11/15 (73%, 95% CI 44.9 to 92.2%)	50–69%	–	< 50%	–
7	Retention in trauma-specific mindfulness-based cognitive therapy group (intervention arm)	Proportion of participants who received at least four sessions (out of those who started the group)	≥ 60%	11/11 (100%)	40–59%	–	< 40%	–
8	Acceptability of trauma-specific mindfulness-based cognitive therapy (intervention arm)	Qualitative process evaluation	Most participants find acceptable or only minor amendments needed.	–	Conflicting views or larger changes needed	Interviewees suggested safety measures, amendments to course content, home practice.	Most participants find unacceptable	–
9	Acceptability of data collection methods	Qualitative process evaluation	Most participants find acceptable or only minor amendments needed	Most participants found data collection tools and procedures acceptable.	Conflicting views or larger changes needed	–	Most participants find unacceptable	–

Between August 2019 and February 2020, 17 trial participants gave permission to be contacted for the process evaluation interviews, and 11 consented to participate in face-to-face interviews (9 from the TS-MBCT arm and 2 from the control arm).

#### Feasibility outcomes

##### Recruitment

**Fig. 2:**
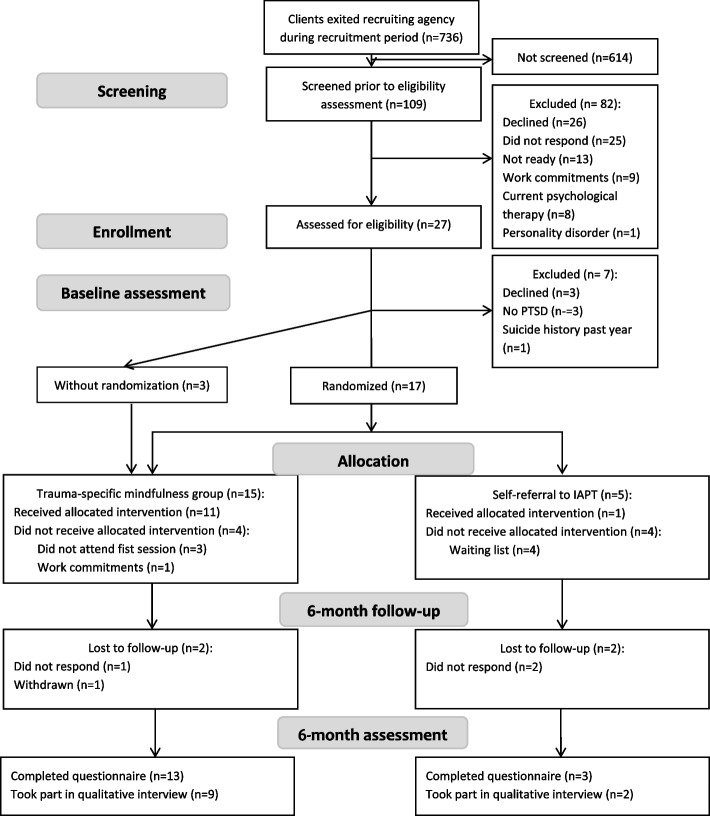
CONSORT diagram. PTSD—post-traumatic stress disorder. IAPT—Improving Access to Psychological Therapies services on the National Health Service

We met our recruitment progression criteria at the amber level. Between February and September 2019, 20 women consented to take part in the trial. Of these, 12 were recruited during the two 3-month time-periods prior to the TS-MBCT groups. Of the four collaborating DVA agencies, only one referred to the trial. Between January and August 2019, agency workers screened 15% of their clients (109/736) and referred 78 to the researcher. After four contact attempts, 25 women did not respond and 26 declined to take part. The main reasons were unreadiness to engage in a psychological therapy (*n* = 13) and work commitments (*n* = 9). A study researcher assessed the eligibility for 27 women, and recruited 20. Due to slow recruitment, we had to re-schedule the first TS-MBCT group twice, and use a third buffer month to fill both groups. To allow extra time for trial set-up, and to fill the first TS-MBCT group, we followed advice from the Study Steering Committee and paused randomisation to the first group for a month. We recruited seven women into the first TS-MBCT group (four with randomisation and three without), and eight women into the second TS-MBCT group (all randomised); five women were randomised into self-referral to the NHS psychological support service (Fig. 2).

The recruitment ratio of 18.3% (20/109 95% CI 11.6% to 26.9%) was lower than the target of 30% [25]. The seven women who were assessed for eligibility but not recruited had a mean age of 38.6 years, and white ethnic background. These characteristics are similar to the 20 recruited women regarding age (mean age of 40.2); however only 75% were of a white ethnic background. The 15 women in the intervention arm had a mean age of 37.9 years, and the five in the control arm 47.2 years (Table 5).

**Table 5:** Baseline characteristics of women allocated to intervention and control arms of the feasibility trial

Characteristic	Intervention *n*/15 (%)	Control *n*/5 (%)	Total, *n*/15 (%)
Age median (IQR)	37 (29–48)	46 (46-54)	40.5 (30.5–49)
Age mean (SD, range)	37.9 (10.4; 20–52)	47.2 (10.6;31–59)	40.2 (10.9, 20–59)
Ethnicity :			
- White: British/Eastern European/Other	11/15 (73.3%)	4/5 (80.0%)	15/20 (75.0%)
- Mixed/Multiple Ethnic Background	1/15 (6.7%)	0/5 (0%)	1/20 (5.0%)
- Asian/Asian British	0/15 (0%)	1/5 (20.0%)	1/20 (5.0%)
- Black/African/Caribbean/Black British/Other Black	2/15 (13.3%)	0/5 (0%)	2/20 (10.0%)
- Other ethnic group	1/15 (6.7%)	0/5 (0%)	1/20 (5.0%)
The highest level of education completed:			
- Primary school	2/15 (13.3%)	0/5 (0%)	2/20 (10.0%)
- Secondary/High school	3/15 (20.0%)	0/5 (0%)	3/20 (15.0%)
- College/University	9/15 (60.0%)	5/5 (100.0%)	14/20 (70.0%)
- Postgraduate degree	1/15 (6.7%)	0/5 (0%)	1/20 (5.0%)
Work status over the last 12 months:			
- Employed/self-employed	3/15 (20.0%)	1/5 (20.0%)	4/20 (20.0%)
- Looking after home/family	4/15 (26.7%)	2/5 (40.0%)	6/20 (30.0%)
- Unemployed and looking for wok	1/15 (6.7%)	0/5 (0%)	1/20 (5.0%)
- Unable to work due to sickness or disability	7/15 (46.7%)	2/5 (40.0%)	9/20 (45.0%)
Marital status:			
- Married	1/15 (6.7%)	1/5 (20.0%)	2/20 (10.0%)
- Living as a couple	1/15 (6.7%)	0/5 (0%)	1/20 (5.0%)
- Divorced or separated	6/15 (40.0%)	4/5 (80.0%)	10/20 (50.0%)
- Single	7/15 (46.7%)	0/5 (0%)	7/20 (35.0%)
Parent	11/15 (73.3%)	4/5 (80.0%)	15/20 (75.0%)
Age of children (0 if not a parent):			
- Age 0–7	4/12 (33.3%)	2/3 (66.7%)	6/15 (40.0%)
- Age 8–18	6/12 (50.0%)	2/3 (66.7%)	8/15 (53.3%)
- Over 18 years old	5/12 (41.7%)	3/4 (75.0%)	8/16 (50.0%)
Housing situation:			
- Homeowner	3/15 (20.0%)	1/5 (20.0%)	4/20 (20.0%)
- Tenant	8/15 (53.3%)	2/5 (40.0%)	10/20 (50.0%)
- Living with relative/friend	2/15 (13.3%)	0/5 (0%)	2/20 (10.0%)
- Other	2/15 (13.3%)	2/5 (40.0%)	4/20 (20.0%)
Financial situation:			
- Living comfortably	1/15 (6.7%)	0/5 (0%)	1/20 (5.0%)
- Doing alright	2/15 (13.3%)	1/5 (20.0%)	3/20 (15.0%)
- Just about getting by	9/15 (60.0%)	2/5 (40.0%)	11/20 (55.0%)
- Finding it difficult to make ends meet	2/15 (13.3%)	2/5 (40.0%)	4/20 (20.0%)
- Finding it very difficult to make ends meet	1/15 (6.7%)	0/5 (0%)	1/20 (5.0%)
Childhood trauma, total score median (IQR)	81 (52–108)^a^	51 (48–56)	65 (51–105)
Childhood trauma, total score mean (SD, range)	76.1 (28.2; 38–111)^a^	49.0 (23.5; 31–90)	67.1 (29.1; 31–111)
- Emotional abuse score mean (SD, range)	16.9 (5.2; 9–25)	11.2 (7.4; 5–24)	15.5 (6.2; 5–25)
- Physical abuse score mean (SD, range)	11.5 (6.6; 5–22)^b^	7.4 (5.4; 5–17)	10.4 (6.4; 5–22)
- Sexual abuse score mean (SD, range)	12.8 (8.0; 5–25)	9.2 (5.5; 5–18)	11.9 (7.5; 5–25)
- Emotional neglect score mean (SD, range)	16.8 (6.4; 7–25)^c^	13.6 (6.9; 7–24)	15.8 (6.5; 7–25)
- Physical neglect score mean (SD, range)	11.8 (4.0; 5–18)^d^	7.6 (3.6; 6 - 14)	10.7 (4.2; 5–18)
Childhood trauma (dichotomised scale scores)			
- Emotional abuse	11/15 (73.3%)	1/5 (20.0%)	12/20 (60.0%)
- Physical abuse	7/14 (50.0%)	1/5 (20.0%)	8/19 (42.1%)
Sexual abuse	9/15 (60.0%)	2/5 (40.0%)	11/20 (55.0%)
- Emotional neglect	6/13 (46.2%)	2/5 (40.0%)	8/18 (44.4%)
- Physical neglect	10/14 (71.4%)	1/5 (20.0%)	11/19 (57.9%)
- Minimisation/denial	1/14 (7.1%)	1/5 (20.0%)	2/19 (10.5%)
Currently afraid of her partner	0/15 (0%)	0/5 (0%)	0/20 (0%)
Ever been afraid of her partner	11/15 (73.3%)	5 /5(100%)	16/20 (80.0%)
Intimate partner violence in past 12 months, total score median (IQR)^b^	12 (2-21)^e^	41 (25–44)	19 (5-25)
Intimate partner violence in past 12 months, total score mean (SD, range)	13.0 (12.4; 0–41)^e^	32.9 (15.3; 9.6–45)	18.5 (15.7; 0-45)
Received support for domestic violence and abuse			
- Advocacy individual	7/11 (63.6%)	4/4 (100.0%)	11/15 (73.3%)
- Freedom programme group	9/12 (75.0%)	3/4 (75.0%)	12/16 (75.0%)
- Recovery Toolkit group programme	1/11 (9.1%)	0/4 (0%)	1/15 (6.7%)
- Peer support group	3/11 (27.3%)	0/4 (0%)	3/15 (20.0%)
- Other	1/11 (9.1%)	0/4 (0%)	1/15 (6.7%)
Ever experienced mental health problems	15/15 (100.0%)	5/5 (100.0%)	20/20 (100.0%)
Ever sought professional help for mental health problems	14/15 (93.3%)	5/5 (100.0%)	19/20 (95.0%)
Ever prescribed treatment for mental health problems (0 if never sought professional help)			
- Self-help materials	5/15 (33.3%)	2/5 (40.0%)	7/20 (35.0%)
- Talking therapy	14/15 (93.3%)	3/5 (60.0%)	17/20 (85.0%)
- Antidepressants	12/15 (80.0%)	4/5 (80.0%)	16/20 (80.0%)
- Other	4/13 (30.8%)	1/5 (20.0%)	5/18 (27.8%)

Frequencies are reported as* n*/*N* (%) unless otherwise stated. n number of completed questionnaires, *N* number of participants in the trial arm

^a^Five missing scores not all questions completed

^b^Two missing scores not all questions completed

^c^Three missing scores not all questions completed

^d^One missing score not all questions completed

^e^Two missing scores not all questions completed

The qualitative process evaluation found that most women thought that the recruitment procedure was acceptable. Interviewees suggested optimising recruitment by targeting women who do not want to take medications for their mental health problems, and women with stable housing and jobs. They suggested extending recruitment to community mental health services, general practitioners, health visitors, women peer support groups, and providers of legal services for women who have experienced DVA. The women also suggested a trauma-specific recruitment process to ensure that potential participants feel safe:

“a lot of survivors still live in fear, we need to help them overcome their fear, -am I going to be victimised by this organisation?” (Participant 1).

One woman found that the time lag between recruitment and intervention was too long, and recommended a connecting call from the teacher, to foster a stronger sense of safety. More signposting for ethnic minority women was recommended.

##### Randomisation

We met our randomisation progression criteria at the amber level. Of the 11 interviewees, 2 women reported not remembering or understanding the randomisation procedure and suggested better explanation. Participants felt that randomisation into the NHS psychological support service arm was not appealing:

“If I were randomized into IAPT arm [NHS psychological support service ‘Improving Access to Psychological Therapies’], I would not stay in the study because I tried everything within IAPT.” (Participant 2)

##### Follow-up

We met our follow-up progression criteria at the green level, with 16 out of 20 women (80%) providing primary outcome data at 6 months post-randomisation. In total, four women were lost to follow-up (LTFU), two per trial arm (Fig. 2). The mean age of the four women LTFU was 33.5 (SD 10.9) compared to 41.9 (SD 10.6) in those who provided primary outcome data. At baseline, all four women LTFU had PTSD according to the PTSD checklist for DSM-V (PCL-5) [36], and 2 (50%) had CPTSD according to the International Trauma Questionnaire (ITQ) [37]. This compares to 14/16 (87.5%) with PTSD according to the PCL-5 checklist, and 2/16 (12.5%) with PTSD and 11/16 (68.75%) with CPTSD according to the ITQ at baseline. The average Composite Abuse Scale Revised-Short Version (CASR-SF) [41] Intimate partner violence scores were 20.5 (SD 17.4) for those LTFU and 17.9 (SD 15.9) for those who provided outcome data. Process evaluation interviews found that for some women the questions asked at follow-up could be triggering:

“I felt quite unsettled after, I guess, because you're asked all these questions.” (Participant 3)

##### Uptake of TS-MBCT

We met our intervention uptake criteria at the green level. Of the 15 women allocated into TS-MBCT arm, 11 started the group (73%, 95% CI 44.9 to 92.2%) which is better than the 33% we were expecting to drop out between randomisation and the start of the mindfulness group. Considering the number of women who never started the mindfulness group, the recruitment ratio is actually 14.7% (16/109, 95% CI 8.6 to 22.7%)—about half of our original estimate [25].

##### Uptake of control interventions

Process evaluation interviews and NHS data suggested that self-referral to the NHS psychological support service would be unfeasible as a control intervention in a full-size trial due to long waiting lists and variability in provided therapies. Of the five women randomised into the control arm, only one was new to the NHS service, self-referred post-randomisation, and was on a waiting list for therapy at 6-month follow-up. The other four women had already been NHS service patients. One woman did not disclose that she was already attending an NHS group therapy for PTSD. Another woman had already received four courses of NHS therapies for anxiety and PTSD and did not self-refer post-randomisation. A third woman had received NHS therapy for anxiety and depression in the past and did not self-refer post-randomisation. And the fourth woman was already on the NHS waiting list before randomisation. At 6-month follow-up, she was still on the waiting list.

##### Retention in TS-MBCT group

We met our intervention retention criteria at the green level. All 11 (100%) women who started a TS-MBCT group attended four or more group sessions. Two women missed one session only, and one woman missed two sessions.

##### Acceptability of TS-MBCT intervention

We met our intervention acceptability criteria at the amber level. On a scale of 1 to 10, eight women scored the importance of their learning journey at 10, one at 9, and one at 8. Five of the 10 women felt that home practice activities and filling in home practice diaries were “the least helpful and/or least enjoyable thing about the course”. The process evaluation interviews found two factors that were important to women’s sense of safety. First, that they knew in advance who the other participants in their group were. Second, that on the first week, the mindfulness teacher met each woman as they arrived at the building entrance when anxiety levels were heightened. One woman recommended moving the ‘nourishing and depleting exploration’ (from week 7) to an earlier week in the course, to encourage home practice as part of selfcare. Some women stated a preference for the body scan practice to be introduced later in the 8-week sequence, once women had learnt how to respond to “side effects” of the practice:

“…always [becoming] aware of certain parts of body broken and hurt by [the] perpetrator during [the] body scan [practice]. Felt very cautious including those parts of the body…I felt I always had a choice…but during home practice a couple of times I had a flashback of perpetrator’s aggressive face but I was able to ground myself and come back to the breath and self soothe with words...” (Participant 4).

Although shorter practices are often recommended for trauma-sensitive mindfulness [59], two women reported needing longer practices because hyper-vigilant states meant it took longer to settle, making shorter practices “challenging” to experience (Participant 4). One woman reported needing more guidance and supportive materials for the mindful movement practices to support recall. To address women’s suggestions for optimising home practice, we developed a coMforT app and tested it with the study advisory group.

##### Data collection methods

We met our data collection methods criteria at the green level. Questionnaire responses and process evaluation interviews showed that most participants found data collection tools and procedures acceptable. The primary candidate outcome (PTSD/CPTSD) questionnaires had 6.3% (1/16) of missing data for the PCL-5 total score and the ITQ Disturbances in Self-Organization (DSO) score, whilst all others were 100% complete. In both cases, one question was left unanswered, and the completed questions allowed us to determine the outcomes for the categorised versions of these questionnaires. Within the secondary candidate outcome questionnaires, 6.3% (1/16) were missing one answer within the Patient Health Questionnaire-9 (PHQ-9), 12.5% (2/16) were missing one answer within the Generalised Anxiety Disorder-7 (GAD-7) questionnaire, and 12.5% (2/16) of participants did not complete any of the CASR-SF intimate partner violence questionnaire—one of whom had experienced childhood abuse, and had never had an intimate adult relationship.

##### Adverse events

We registered two adverse events in the TS-MBCT arm. One woman, who was living with the perpetrator of the abuse, reported that attending the group had increased her anxiety. She reported that the body scan practice had increased feelings of self-hatred towards her body, particularly when the body scan was practiced at home. Another woman reported increased anxiety after realising that the daughter of a man who assaulted her was attending the same group. Both women received additional support from the teacher, and one had additional support from her general practitioner and engaged with counselling.

##### Candidate clinical outcomes in full trial

We found that the measures of the primary outcomes (PTSD/CPTSD) at screening and assessment need further refinement. Although all 20 women scored ≥ 3 on the PC-PTSD-5 [33] at final screening, some did not score above an established threshold for PTSD/CPTSD on the PCL-5 [36] and ITQ [37] at baseline. The PCL-5 measured PTSD constructs from the Diagnostic and Statistical Manual of Mental Disorders, 5th Edition (DSM-5) [60], while the ITQ measured different PTSD/CPTSD constructs from the International Classification of Diseases 11th Revision (ICD-11) [61]. It appears that the ITQ identified less people with clinically important symptoms, with the ‘re-experiencing’ criteria an important determinant. At baseline, 18/20 (90%) scored above the PTSD threshold on the PCL-5 whilst only 15/20 (75%) scored for either PTSD (*n* = 2) or CPTSD (*n* = 13) on the ITQ. At 6 months, fewer individuals self-reported PTSD symptoms on the ITQ. We have summarised candidate primary and secondary outcomes for a fully powered trial in Additional file 5.

At the 6-month follow-up, none of the 95% CIs ruled out a clinically important difference in candidate outcomes between trial arms, indicating that it is reasonable to proceed to a full-size RCT to estimate these outcomes with greater precision. Overall, PCL-5 scores were lower in the intervention arm with a mean difference of − 16.6 (95% CI − 42.3 to 9.1) and both ITQ PTSD and DSO scores were also lower in the intervention arm with a mean difference of − 5.5 (95% CI − 14.4 to 3.3) and − 7.4 (95% CI − 16.6 to 1.7) respectively (Table 6).

**Table 6:** Potential effect on candidate clinical outcomes at 6-month follow-up

Outcomes at 6-month follow-up	Intervention (*n* = 13)		Control (*n* = 3)		Mean or risk difference: intervention–control (95% CI)
	*N*	Mean (SD) or *n* (%) if stated	*N*	Mean (SD) or *n* (%) if stated	
Primary outcomes					
PTSD Checklist DSM-5 score	12	30.8 (18.0)	3	47.3 (20.8)	− 16.6 (− 42.3 , 9.1)
PTSD Checklist DSM-5 *n* (%) above PTSD threshold	13	8 (61.5%)	3	2 (66.7%)	− 0.05 (− 0.65, 0.54)
International Trauma Questionnaire–PTSD score	13	8.5 (6.8)	3	14.0 (3.6)	− 5.5 (− 14.4, 3.3)
International Trauma Questionnaire–DSO score	12	9.9 (6.6)	3	17.3 (6.4)	− 7.4 (− 16.6, 1.7)
International Trauma Questionnaire *n* (%) above threshold:					
- PTSD	13	1 (7.7%)	3	0 (0%)	0.08 (− 0.07, 0.22)
- CPTSD	13	2 (15.4%)	3	0 (0%)	0.15 (− 0.04, 0.35)
Secondary outcomes					
Dissociative symptoms, DES-B	13	7.8 (5.3)	3	11.3 (6.7)	− 3.6 (− 11.2, 4.0)
Depression, PHQ-9	13	9.2 (5.2)	2	21.5 (3.5)	− 12.3 (− 20.5, − 4.0)
Anxiety, GAD-7	12	6.6 (3.8)	2	12.5 (7.8)	− 5.9 (− 13.0, 1.2)
Self-compassion	13	3.0 (0.8)	3	2.3 (0.6)	0.8 (− 0.3, 1.8)
Intimate partner violence score in past 6 months	12	3.6 (4.1)	2	10.5 (2.1)	− 6.9 (− 13.5 , -0.4)
Health economic outcomes					
Quality of life, women, EQ-5D-5L	13	0.57 (0.28)	3	0.31 (0.458)	0.26 (− 0.17, 0.69)
Quality of life, index child, KIDSCREEN-10	4	53.8	0	–	–

*N* completed questionnaire, *SD* Standard deviation, *CI* Confidence interval, *PTSD* Post-traumatic stress disorder, *CPTSD* Complex post-traumatic stress disorder, *DSM-5* Diagnostic and Statistical Manual of Mental Disorders, 5th Edition, *PHQ-9* Patient Health Questionnaire, *GAD-7* Generalised Anxiety Disorder questionnaire, *EQ-5D-5L* health-related quality of life states in adults questionnaire, *KIDSCREEN-10* health-related quality of life questionnaire for children

At 6 months, the proportion of participants who scored above the PTSD threshold on the PCL-5 were also lower in the intervention arm with a risk difference of − 0.05 (95% CI − 0.65 to 0.54); however, the proportion of those who scored above PTSD and CPTSD thresholds on the ITQ were higher in the intervention arm with a risk difference of 0.08 (95% CI − 0.07 to 0.22) and 0.15 (95% CI − 0.04 to 0.35) respectively. The point estimates for all the secondary outcomes were better within the intervention arm, but with wide confidence intervals.

##### Health economic evaluation feasibility

Of the 20 participants, 16 (80%) completed the resource use questionnaire at 6 months. Resource use occurred across several non-NHS services including criminal justice. Individual questions of the bespoke resource-use questionnaire were well completed, but some questions had no responses. Both emergency department and outpatient use was high, with 44% and 50% respectively using these services over the 6-month period. Intervention resource use captured a total therapist time of 41.5 h per TS-MBCT group.

All 20 participants completed baseline EQ-5D-5L, with 16 (80%) of participants completing EQ-5D-5L at follow-up. Quality of life scores at baseline and follow up were well below the UK adult general population of 0.856 [62]. Mean baseline EQ-5D-5L across the sample was 0.474 (SD 0.302) with a maximum score of 1 (one participant) and a minimum of − 0.046. Mean follow up EQ-5D-5L was slightly higher at 0.520 (SD 0.320) with a maximum score of 0.837 and minimum of − 0.218. Of the 20 women, 44% had children. Women completed KIDSCREEN-10 quality of life questionnaires [45] for 5 children at baseline and 4 at follow-up (Table 6).

## Discussion

### Main findings

In collaboration with experts in trauma and mindfulness and women with lived experience, we developed a DVA-specific TS-MBCT intervention for the treatment of PTSD/CPTSD. Our evidence synthesis on trauma-specific adaptations to standard mindfulness programmes identified gaps and conflicting evidence on psychological process, exclusion criteria, teacher characteristics, and teacher-participant relationships dynamics that are specific to the DVA population. The consensus exercise with experts in trauma and mindfulness helped fill the gaps and clarify uncertainty in the evidence. Our theory-driven, evidence-based, pragmatic approach to intervention refinement produced a TS-MBCT intervention that was found to be acceptable to women with a history of DVA. We recruited 20 women into a feasibility trial (15 into a TS-MBCT arm, and 5 into a NHS psychological therapy arm) and retained 80% at 6 months (87% intervention vs 60% control). Eleven participants found the trial procedures acceptable and suggested additional recruitment pathways. Some participants suggested clarifying the randomisation procedure and some felt that randomisation into the non-TS-MBCT arm was unfavourable. Intervention uptake was 73% (95% CI 44.9 to 92.2%), and retention was 100%. We registered two adverse events in the TS-MBCT arm. Some participants suggested additional safety measures and minor amendments to the course content and home practice. Most participants found the data collection procedure for candidate clinical outcomes for a full trial acceptable. However, the questionnaires for PTSD/CPTSD screening (PC-PTSD-5) and assessment (PTSD Checklist DSM-5, ITQ) showed differing frequencies of clinically important PTSD/CPTSD symptoms. At 6 months, none of the 95% CIs ruled out a clinically important difference in candidate outcomes, indicating that it is reasonable to proceed to a full-size RCT to estimate these outcomes with greater precision. We will use the SD of the candidate clinical outcome measures to inform a sample size calculation for a full trial. The feasibility trial met our pre-specified progression criteria at the green (*n* = 6) and amber (*n* = 3) light targets. The fully powered RCT of the TS-MBCT intervention was given the amber light to proceed after making amendments to the recruitment and randomisation procedures, control intervention, primary outcomes measures, and intervention content.

In line with previous research with this population [63], we faced challenges with timely recruitment. Despite regular site visits, only one out of four DVA agencies referred potential participants to the study. In a full trial, we will broaden the scope of recruitment to multiple specialist DVA agencies, general practice, community mental health services, health visitors, peer support groups, and other services for women who have experienced DVA. Recruitment sites should be screening more service users to identify enough potential trial participants. More targeted recruitment of Black, Asian, and minority ethnic women is recommended to increase diversity and inclusivity of the sample population. The full trial should include an internal pilot to test the feasibility and acceptability of the suggested additional recruitment pathways and procedures.

Uncertain acceptability of the randomisation process for some of our participants has implications for the design of the full trial. Misunderstanding of randomisation should be addressed through explaining its implications and the concept of clinical equipoise in plain English. Another reason for low acceptability may have been the self-referral to the NHS psychological support service as a control intervention, since the long waiting lists and prior negative experiences are regarded as inferior. Having an active evidence-based psychological control intervention could improve the acceptability of randomisation.

The high acceptability of trial procedures and the TS-MBCT intervention is consistent with prior studies [51, 52, 63]. It could be improved by providing a safety protocol for any women triggered by questionnaire items, maintaining contact between recruitment and start of the intervention, and managing pre-intervention anxiety with a group orientation session. Orientation sessions can offset both anxieties related to travelling, and arriving at a new location and unfamiliar building, as well as manage fears about encountering someone connected to the perpetrator or a woman’s community. A protocol is needed that can be shared with women at the orientation session to explain how the teacher and group participants will manage confidentiality and safety concerns. A minority of women in the process evaluation recommended longer practices to accommodate barriers to settling attention, such as hyper vigilance. A minority of women recommended moving the sequencing of key practices, again to support a sense of safety and increase capacity to manage ‘side effects’ of practices such as the body scan.

Another area for improvement is the tools for PTSD/CPTSD screening and assessment. Although data collection for the primary outcomes was complete, we found that some women who screened positive on the PC-PTSD-5 [33], did not score above the PTSD/CPTSD threshold on the PCL-5 [36] and ITQ [37]. In the full trial, we will choose a more specific screening tool for PTSD, and use a clinician administered assessment after initial screening [64, 65] to ensure all trial participants have PTSD/CPTSD diagnosis at baseline.

Health economic data showed that our participants had high levels of resource use outside of health care, particularly in the criminal justice system. Therefore, we need a societal perspective analysis alongside a health and social care analysis in the full trial. EQ-5D-5L and KIDSCREEN data were successfully collected and sensitive to changes in quality of life. Limited evidence of floor or ceiling effects means a cost-utility-analysis presenting cost-per-QALY will be feasible in a full trial.

### Strengths and limitations

We followed evidence-based guidance on developing health care interventions and involved people with lived experience and professional stakeholders from the start and throughout the intervention refinement process and feasibility testing. This systematic approach produced a prototype intervention and trial procedures that are likely to be feasible and acceptable to women with PTSD/CPTSD and a history of DVA. We pilot-tested the prototype TS-MBCT course twice and evaluated the intervention acceptability after the first group. Findings from the interim process evaluation informed refinement of the intervention and trial procedures for the second TS-MBCT group. This iterative process contributed to the development of a more acceptable intervention.

The coMforT feasibility trial targeted women with PTSD/CPTSD and a history of DVA, a population that is challenging to engage safely in research. Women who have experienced DVA must first address their basic needs, and those of their children (e.g. safety, housing, childcare), before considering activities for their own health and wellbeing, including engaging in research [66]. We facilitated women’s engagement by recruiting through a DVA agency, which provided risk assessment, specialist advocacy, and assistance with addressing basic needs. By recruiting at service exit, we ensured that all participants had received specialist support by the time of trial recruitment. This pathway, alongside safeguarding during the trial, minimised harm from the intervention and research procedures.

A limitation of the coMforT study is the recruitment via only one DVA agency, which did not allow us to explore different settings. A future full-size trial should aim to recruit across diverse settings and sectors to increase generalisability and enrol more women from ethnic minorities. Further limitations include low number of participants and lack of randomisation for some participants at the start of the trial. These should be addressed through optimising recruitment and randomisation procedures.

## Conclusion

The coMforT study produced a TS-MBCT intervention that addresses specific vulnerabilities of women with clinically important symptoms of PTSD/CPTSD and a history of DVA. It is feasible to conduct a full-size trial of our TS-MBCT intervention versus active group psychological treatment for PTSD/CPTSD in the DVA population. Our TS-MBCT intervention had high uptake and retention. Slow recruitment from a single DVA agency indicates that future recruitment should be extended to multiple DVA agencies, NHS, and non-NHS settings to identify and approach enough women with PTSD/CPTSD and history of DVA who feel ready and are able to engage with a group psychological therapy.

## Supplementary Information

**Additional file 1: Supplementary file 1. Phase 1.** Interview topic guides.

**Additional file 2: Supplementary file 2. Phase 1.** Online survey with experts in trauma and mindfulness.

**Additional file 3: Supplementary file 3. Phase 2.** Process evaluation interview topic guide.

**Additional file 4: Supplementary file 4. Phase 1.** Evidence matrix.

**Additional file 5: Supplementary file 5.** Raw candidate clinical outcomes at baseline and 6-month follow-up.

## Data Availability

Due to the sensitivity of the data involved, these data are published as a controlled dataset at the University of Bristol Research Data Repository data.bris, at https://doi.org/10.5523/bris.j4jsx8boace42hg58jcvyinlo. The metadata record published openly by the repository at this location clearly states how data can be accessed by bona fide researchers [68]. Requests for access will be considered by the University of Bristol Data Access Committee, who will assess the motives of potential data re-users before deciding to grant access to the data. No authentic request for access will be refused and re-users will not be charged for any part of this process.

## Abbreviations

AUDIT-C: Alcohol Use Disorders Identification Test Consumption
CI: Confidence interval
coMforT: Mindfulness for Trauma study
CPTSD: Complex post-traumatic stress disorder
DSM-5: Diagnostic and Statistical Manual of Mental Disorders 5th Edition
DUDIT: Drug use Disorders Identification Test
DVA: Domestic violence and abuse
EMDR: Eye movement desensitisation and reprocessing
GAD-7: Generalised Anxiety Disorder
ICD-11: International Classification of Diseases 11th Revision
ITQ: International Trauma Questionnaire
LEC-5: Life Events Checklist for DSM-5
MBCT: Mindfulness-based cognitive therapy
NHS: National Health Service
OR: Odds ratio
PCL-5: PTSD Checklist for DSM-5
PC-PTSD-5: Primary Care PTSD Screen for DSM-5
PHQ-9: Patient Health Questionnaire
PTSD: Post-traumatic stress disorder
RCT: Randomised controlled trial
TF-CBT: Trauma-focused cognitive behavioural therapy
TS-MBCT: Trauma-specific mindfulness-based cognitive therapy
UKPTS: United Kingdom Psychological Trauma Society
