# Supplementary material for: Trauma-specific mindfulness-based cognitive therapy for women with post-traumatic stress disorder and a history of domestic abuse: intervention refinement and a randomised feasibility trial (coMforT study)
Source: Pilot Feasibility Stud. 2023 Jul 3;9:112. doi: 10.1186/s40814-023-01335-w (PMC10316568; doi:10.1186/s40814-023-01335-w)
Supplement: Supplementary file 1 — Additional file 1: Supplementary file 1. Phase 1. Interview topic guides. [file 40814_2023_1335_MOESM1_ESM.docx]

# Supplementary file 1. Phase 1. Interview topic guides

## Professional interview topic guide

1. Could you start by telling me a bit about your current role in relation to survivors of domestic violence and abuse and/or service users experiencing symptoms of trauma:

- - What are your experiences regarding this client group?
  - In your experience, what are the ways in which trauma impacts the lives of the people you are working with?
  - What might their specific needs be?

2. Explore their views about therapeutic treatments available for survivors of domestic violence and abuse (DVA) with trauma symptoms/post-traumatic stress disorder (PTSD):

- - What types of therapeutic approach are you aware of to support survivors of DVA with PTSD?
  - What do you think about the various approaches?
  - Which ones have you practiced/provided/commissioned/recommended?
  - What attracted you to this approach? Which do you think are the most important parts of the treatment?
  - What do you think are the main barriers and facilitators to people who might benefit from this type of therapeutic treatment?
  - Which approaches do you think give positive outcomes? What are those positive outcomes?
  - What do you think are the short-term/long-term benefits of these approaches?

3. Explore beliefs and experiences of mindfulness as a therapeutic treatment:

- - What do you know about mindfulness as a therapeutic approach?
  - Is it an approach you’ve practiced/provided/commissioned/ recommended?
  - Is it an approach you’ve considered? Explore reasons why/ why not

If they have practiced/provided/commissioned/recommended it:

- - what attracted you to this approach?
  - what did you hope service users might gain?
  - what do you think are the most important parts of this therapeutic treatment?
  - what changes and impacts do service users notice? (good and bad, short- and long- term - including on-going use of techniques)

If they have not practiced/provided/commissioned/recommended it:

- - have you practiced/provided/commissioned/recommended a different approach instead?
  - do you have any concerns about a mindfulness-based approach? If so, what are your concerns?
  - do you see any barriers to a mindfulness-based approach? If so, what do you think the barriers are?
  - what would make you more likely to practice/provide/commission/recommend this approach?

4. In the next stage of this project, we will be inviting women who have experienced domestic violence to join a mindfulness group.

Give them description of coMforT prototype intervention to read. Thinking about what we are proposing, what are your initial thoughts?

If we go through section by section, could you tell me what you think about:

4.1. The idea of Mindfulness for trauma survivors:

- Interest in this for their client group
- Any hesitations
- Thoughts about feasibility
- Barriers and facilitators to participants
- Overall acceptability to the profession they represent

4.2. The way the course will be run:

- Time (day, time, length, number of sessions?)
- Location (easy to find; convenient; travel; environment; accessibility; confidentiality; type of location - hospital, institution, third sector org. - possibility of stigma?)
- Childcare arrangements
- Being part of a therapist-led group (relationship with therapist, and with other participants – group dynamics, ground rules, leadership)

4.3. Who can attend the course:

- Particularly explore the ineligibility criteria (length of time free from abuse, receiving other therapy, addiction, comorbidities/complex needs)

5. Explore in detail any suggestions, recommendations and refinements they may make about the intervention.

## Service user interview topic guide

1. We’ll move on to talking about different types of support that you may have experienced, but to give us some context, could you start by telling me a bit about the relationship you were in?

- - Find out how long ago the relationship ended.

2. How did you cope, during and after the relationship?

- - Explore coping – (if has kids, what did you/do you do when they’ve gone to bed? What was/is helpful? what was/is unhelpful?)
  - Explore help-seeking
    1. Who have they approached for help (professionals/lay people)?
    2. Who offered support?

3. What kinds of impacts did you experience?

- - Impacts on daily life?
  - Impacts on children?
  - Explore any particular wellbeing and mental health impacts mentioned
  - Explore whether any of these persisted after the end of the relationship
  - Find out whether they are still experiencing symptoms now
  - Explore any trauma symptoms mentioned:

e.g. People who’ve had distressing or horrible experiences in a relationship may experience some trauma symptoms afterwards, have you noticed any yourself? Explore the following:

- - Intrusive, distressing memories of events, flashbacks, nightmares.
  - Emotional numbness and avoidance
  - Difficulty sleeping, jumpiness, emotions close to surface.

4. How have you found managing these impacts?

- - Have they had any therapeutic treatment?

If yes: what type was it? can you describe what kinds of things you did with the therapist? were they offered any choices about treatment? what made them choose this option? what was their experience of it? what did they like about it? what worked well/not so well (at the time and long term)? how did they feel immediately after/between sessions? would they recommend it to other people?

If no: have they considered any therapeutic options? what has put them off pursuing this treatment?

5. Explore beliefs and experiences of mindfulness as a therapeutic treatment

If they’ve had a mindfulness-based treatment or practised mindfulness themselves:

- - what form did the treatment take? (a course, mindfulness in one-to-one therapy, learning by reading a book and using a CD, an app?)
  - what attracted you to this approach?
  - what did you hope to gain?
  - what were the most important parts of the treatment for you?
  - what did you like/not like about the treatment?
  - have you continued with practising mindfulness?
  - what challenges, if any, have you experienced?
  - what changes and impacts have they noticed? (good and bad)

If they’ve not had a mindfulness-based treatment or practised mindfulness themselves:

- - have they heard of mindfulness?
  - what do they think it is?
  - is it something they’ve considered trying? Explore reasons why not
  - do they know others who have learnt and used mindfulness practices?
  - what do they think other people hope to gain from this approach?

6. In the next stage of this project, we will be inviting women who have experienced domestic violence to join a mindfulness group.

Give them description of intervention on card to read). Thinking about what we are proposing, what are your initial thoughts?

And if we go through section by section, could you tell me what you think about:

6.1. The idea of Mindfulness for trauma survivors:

- Any hesitations
- Thoughts about feasibility
- Barriers and facilitators to women
- Overall acceptability to survivors

6.2. The way the course will be run:

- Time (day, time, length, number of sessions)
- Location (easy to find; convenient; travel; environment; accessibility; confidentiality and knowing other people in the group; type of location - hospital, institution, third sector org. - possibility of stigma)
- Childcare arrangements
- Being part of a therapist-led group (relationship with therapist, and with other participants – group dynamics, ground rules, leadership).

6.3. Who can attend the course:

- Particularly explore the ineligibility criteria (length of time free from abuse, receiving other therapy, addiction, comorbidities/complex needs)

6.4. Explore in detail any suggestions, recommendations, and refinements they may make about the intervention.

## Prototype intervention description

**What is Mindfulness?**

Mindfulness is a process of bringing our attention to experiences happening in the present moment – being aware of where we are, what we’re doing and how we’re feeling. It helps us to recognise when we might be feeling overwhelmed by what is going on – around us or inside us. Mindfulness also helps us to see when we’re worrying about the past or fretting about the future, and to learn how to shift our focus back to the ‘here and now’. Mindfulness supports us to take care of ourselves when life is difficult or when we’re unwell, so that we’re able to respond to ourselves in a kinder way.

**Mindfulness for trauma**

This is a new course which has been adapted to help women survivors of domestic violence and abuse who are experiencing problems with their mental health and wellbeing because of the trauma they’ve been through. During the course, we will be focussing on what’s happening in the ‘here and now’ - this can be especially helpful for trauma survivors, because there is no need to ‘re-live’ experiences from the past. Each session includes time for:

- practising mindfulness in a group
- exploring how the mind works and how mindfulness can help
- learning how to recognise negative thoughts and replace them with positive responses

**Course practicalities**

- The course will last for 8 weeks and take place in 2-hour sessions during school hours in a venue which is easily accessible
- There will usually be about 8 women in each group
- Before starting the course, each woman will come along to an information session, where they will complete a simple form and have a chat with the course teacher
- The course is taught by an experienced and qualified mindfulness teacher, who is also a psychotherapist with extra training around trauma
- Women taking part will be given a set of handouts to help reinforce learning and an MP3 player with recordings of mindfulness practices that they will be asked to do at home each day

**Who can attend the course during the study?**

- People can take part in one of the courses if they: are female, are aged 18+, have been receiving support from Next Link, and have symptoms of post-traumatic stress
- Women attending the course will need to be able to read and write in English (because the course and materials will be delivered in English)
- Some people won’t be able to take part in a course, mostly for safety reasons, including: people who have experienced physical or sexual abuse in the past 2 months, people struggling with current drug or alcohol addiction, people who have experienced psychosis, people with brain damage, people at high risk of suicide or active self-harm, and people who are already receiving talking therapy.
