# Supplementary material for: Trauma-specific mindfulness-based cognitive therapy for women with post-traumatic stress disorder and a history of domestic abuse: intervention refinement and a randomised feasibility trial (coMforT study)
Source: Pilot Feasibility Stud. 2023 Jul 3;9:112. doi: 10.1186/s40814-023-01335-w (PMC10316568; doi:10.1186/s40814-023-01335-w)
Supplement: Supplementary file 2 — Additional file 2: Supplementary file 2. Phase 1. Online survey with experts in trauma and mindfulness. [file 40814_2023_1335_MOESM2_ESM.docx]

# coMforT phase 1 consensus survey

## Introduction

This survey includes 15 statements about trauma-informed modifications to a standard MBCT course. DVA - domestic violence and abuse, PTSD - posttraumatic stress disorder. Please score and comment on the statements in a manner which represents your views and experience of teaching mindfulness to trauma survivors. The survey takes on average 30 minutes to complete.

All information you provide will be anonymous and confidential.

You can save your responses part way through and return to the survey later by clicking on the "Finish later" button at the end of each page.

## Participants

What do you see as the key vulnerabilities of the DVA population?

1. More info

- Adverse childhood experiences
- Childhood sexual abuse
- Major depressive disorder co-morbid with PTSD
- Self-harming behaviours intended to manage psychological distress
- Suicidal ideation
- Other

1.a. If you selected Other, please specify:

1.b. How can a trauma-informed MBCT course meet these vulnerabilities?

2. For standard MBCT, in cases of early trauma, past abuse, and dissociative disorders, "mindfulness can be a useful adjunct to psychotherapy ..., but should only be introduced when the client has full support from a therapist who understands mindfulness, and when the client is ready to start making connections with what may be extremely painful material" (https://mindfulnessteachersuk.org.uk/pdf/MBCTImplementationResources.pdf). Our primary study suggests that not all DVA survivors being screened for inclusion may have accessed 1-2-1 support in which they have processed their trauma. Is trauma-informed MBCT suitable as a first line therapy for DVA survivors with PTSD?

- Yes
- No
- Undecided

2.a. Comments

3. Current substance dependence is an exclusion criterion for standard

MBCT (https://mindfulnessteachersuk.org.uk/pdf/MBCTImplementationResources.pdf). However, in the DVA population, PTSD has high co-morbidity with substance misuse. We propose to invite DVA survivors with current substance dependence to the orientation session where

the teacher will assess the survivor's relationship to substance misuse and readiness to participate in an 8-week course.

Strongly disagree Disagree Undecided Agree Strongly agree

3.a. Comments:

4. Persistent self-harm or suicide risk requiring management are exclusion criteria for standard

MBCT (https://mindfulnessteachersuk.org.uk/pdf/MBCTImplementationResources.pdf). However, in the DVA population PTSD has high co-morbidity with self-harm and suicidal ideations. We will use PHQ-9 Q.6.("I have had thoughts I would be better off dead or harming myself in some way in the last two weeks") and the additional question (“I have made plans to end my life in the last two weeks”) to assess suicidality. We propose to invite DVA survivors who score >0 on the above questions to the orientation session where the teacher will assess the survivor's relationship to suicidal ideation and self-harm. The teacher will use professional judgement to assess woman's readiness to participate in an 8-week course.

Strongly disagree Disagree Undecided Agree Strongly agree

4.a. Comments:

5. Standard MBCT manual does not mention English language as inclusion criterion (https://mindfulnessteachersuk.org.uk/pdf/MBCTImplementationResources.pdf). Our primary study suggests that for many DVA survivors English is not their first language. We propose to include DVA survivors who can understand and speak English but may not read or write.

Strongly disagree Disagree Undecided Agree Strongly agree

5.a. Comments:

## Course curriculum & format

6. What is the minimum group size for trauma-informed MBCT?

6.a. Comments:

7. What is the maximum group size for trauma-informed MBCT?

7.a. Comments:

8. The core aim of the standard MBCT is to cultivate a shift from a doing mode to being mode. In our trauma-informed MBCT curriculum, the direction of change in psychological process is conceived as shifting from overwhelm towards cultivating more stability, which offers the possibility of regulating (trauma related) intense internal experience.

Strongly disagree Disagree Undecided Agree Strongly agree

8.a. Comments:

9. Psychoeducational material on depression is included in week 4 of the standard MBCT course. We propose to add psychoeducational material on PTSD in week 4 of the trauma-informed MBCT course. Both materials would be delivered through experiential learning instead of a written handout.

Strongly disagree Disagree Undecided Agree Strongly agree

9.a. Comments:

## Teacher

10. Teachers delivering MBCT to trauma survivors should be trained in trauma-informed MBCT curriculum.

Strongly disagree Disagree Undecided Agree Strongly agree

10.a. Comments:

11. Teachers delivering trauma-informed MBCT should be registered psychotherapists.

Strongly disagree Disagree Undecided Agree Strongly agree

11.a. Comments:

12. Teachers delivering trauma-informed MBCT should have trauma-informed MBCT supervisors (to ensure they have processed their own trauma).

Strongly disagree Disagree Undecided Agree Strongly agree

12.a. Comments:

13. Teachers assistants delivering MBCT to trauma survivors should be trained in trauma-informed MBCT.

Strongly disagree Disagree Undecided Agree Strongly agree

13.a. Comments:

14. Teachers assistants delivering trauma-informed MBCT should also be trained as psychotherapists to adequately meet the vulnerabilities of this group and manage potential individual distress during group sessions.

Strongly disagree Disagree Undecided Agree Strongly agree

14.a. Comments:

15. Our primary study suggests that due to a power imbalance the teacher-participant relationship can mirror the abusive relationship. What needs to be included in training to prevent this?

## About you

16. How long have you been teaching mindfulness to trauma survivors (full years)?
