# Supplementary material for: Trauma-specific mindfulness-based cognitive therapy for women with post-traumatic stress disorder and a history of domestic abuse: intervention refinement and a randomised feasibility trial (coMforT study)
Source: Pilot Feasibility Stud. 2023 Jul 3;9:112. doi: 10.1186/s40814-023-01335-w (PMC10316568; doi:10.1186/s40814-023-01335-w)
Supplement: Supplementary file 3 — Additional file 3: Supplementary file 3. Phase 2. Process evaluation interview topic guide. [file 40814_2023_1335_MOESM3_ESM.docx]

# Supplementary file 3. Phase 2. Interview topic guide

1. Where were you on your pathway to recovery when approached with study invite?

- On the timeline of the abusive relationship (in/out, for how long)
- On the recovery pathway (to what extend processed, what treatments received)
- Re: mental health status
- Readiness

2. Where can we find women survivors, who are potentially ready to enrol in a full-size study?

- Who are the gatekeepers for these potential study participants (e.g., GP, health visitors)?

3. What were your experiences of taking part in the research?

- How did it feel being asked if interested in taking part in the coMforT study (initial response and why?)
- The recruitment processes
- The randomisation process (Understood? Feelings about allocation?)
- Communication with the researchers
- The info given (Enough? Clarity? Format?)
- What has been like to complete the screening, baseline and 6 month questionnaires?
- Would you recommend participating in this, or a similar study to a good friend? If not, why not?

4. How did you find the practical arrangements for the sessions?

- Time (day, time, length?)
- Location (easy to find, convenient, environment, accessibility, confidentiality?)
- Childcare arrangements
- Being part of a therapist-led group/individual sessions (relationship with therapist, and with other participants – group dynamics - for intervention arm).

5. How did you find the approach used in the sessions?

- Had you heard about this/used this type of therapy before?
- How did it compare to previous use/experiences/expectations?
- What worked well? Not so well?
- How did you feel about coming to the sessions?
- What did you like best about the sessions? And least?
- How would you describe your experiences of the sessions to others?
- How did you feel when the sessions ended
- Would you recommend these sessions to other people?

6. Experience of trauma-specific adaptations.

- What was your experience of the body scan? (prompt for any experience of feeling triggered by language used)
- Did you always feel you had the choice to not take part in a practice if it didn’t feel right for you?[experience of invitational language]
- What was it like for you moving out of a practice and back into the group? [experience of transitions]
- What was your experience of the movement practice?
- How did you find the length of the practices?
- Which of the 8 week themes was most helpful for you and why? (e.g. autopilot / recognising aversion/allowing and letting be)
- What was your experience of the written calendar exercises? (pleasant and unpleasant events/relational communication difficulty).

7. How did you find the group format?

- What was it like being a group with other survivors?
- What was it like talking in pairs or talking or listening during group discussions/enquiries?
- Did you find anything particularly helpful or difficult about the group?

8. How did you find the teacher and teacher assistant?

- What were your expectations of the teacher?
- Do you have any suggestions for things the teacher could do differently to improve the experience for other women taking this course?

9. Explore expectations, changes and impacts:

- What were you hoping to gain from the sessions? Was this achieved?
- What did you learn?
  - E.g. how do you view your thoughts now? The tone of your thinking?
  - E.g. did the theme of ‘allowing’ make sense to you?
  - E.g. did the theme of ‘living in our heads’ make sense to you
  - E.g. did the theme of ‘recognising aversion make sense to you?
  - E.g. did the theme of ‘gathering the scattered mind’ make sense to you?
  - E.g. did the theme of ‘thoughts are not facts’ make sense to you?
  - E.g. did the theme ‘how can I best take care of myself’ make sense to you?
  - (prompt for learning on how they understand the relationship between the trauma you experienced, aversive reactions and maintaining distress:)
  - -e.g.do you understand the distress you feel any differently now you have done the course?
- What will you take with you? (Any examples of having put learning into practice?)
- Have you been using any of the practices (e.g.3 minute breathing space feeling your feet on the ground, movement practice ) you learnt since the weekly sessions ended?
- Can you describe any ways in which you feel the sessions have been helpful/unhelpful?
- Have you noticed any impacts whilst/since attending the sessions? (wellbeing, health, mood, coping, help-seeking, interactions with other people, enjoyment, thoughts, abilities - Inc. parenting and work …)

10. Making changes:

- If we were to change or enhance any aspects of the sessions, what would your suggestions be? Add anything? Leave anything out?
