# Supplementary material for: Trauma-specific mindfulness-based cognitive therapy for women with post-traumatic stress disorder and a history of domestic abuse: intervention refinement and a randomised feasibility trial (coMforT study)
Source: Pilot Feasibility Stud. 2023 Jul 3;9:112. doi: 10.1186/s40814-023-01335-w (PMC10316568; doi:10.1186/s40814-023-01335-w)
Supplement: Supplementary file 4 — Additional file 4: Supplementary file 4. Phase 1. Evidence matrix. [file 40814_2023_1335_MOESM4_ESM.docx]

Supplementary file 4. Evidence matrix summarising evidence for trauma-specific adaptations to the standard mindfulness-based cognitive therapy curriculum

| **Trauma-sensitive adaptation** | **Literature review** | **Qualitative interviews with women who have experienced domestic violence and abuse, and with relevant professionals (n=20)** | **Survey with experts in trauma and mindfulness (n=8)** | **Consultation with experts in trauma and mindfulness (n=3)** | **Incorporated into trauma-specific mindfulness-based cognitive therapy curriculum** |
| --- | --- | --- | --- | --- | --- |
| 1. Course curriculum content (adaptations made to content itself, or how aspects of the course are delivered) | | | | | |
| 1.1. Orientation session  Should we have orientation session face-to-face or remotely? Individually or in a group? | Limited evidence.  Individual face-to-face orientation session [56].  Group face-to-face orientation session [55]. | Agreement.  Women and professionals felt that individual orientation was vital to explore key areas, including (i) individual strengths and resources, (internal and external); (ii) specific vulnerabilities and potential triggers (enabling proactive planning). (iii) a shared understanding re course content, structure, expectations, and boundaries; (iv). advance management of expectations; (v). trauma history and processing, abuse context (ongoing contact), wider lifestyle, and needs. | NA | NA | Pragmatic decision to hold individual orientation sessions over the phone. |
| 1.2. Tailoring/tweaking/refining  What is the direction of change in psychological process in the TS-MBCT? | No evidence | NA | Disagreement.  The direction of change in psychological process in the TS-MBCT is conceived as shifting from overwhelm towards cultivating more stability, which offers the possibility of regulating (trauma related) intense internal experiences?  Agree (n=6)  Undecided (n=1)  Disagree (n=1). | Terms and conditions.  The direction of change in psychological process in a TS-MBCT course is consistent with standard MBCT – learning how to be with experience rather than automatically reacting to it. A TS-MBCT course offers additional learning for trauma survivors, however, which includes recognising the ‘window of tolerance’ and discerning between hyperarousal and hypoarousal. This involves developing skills for establishing safety, moving towards challenge with awareness and recognising overwhelm. These skills contribute to cultivating more stability and capacity for regulation of intense experiences. Including psycho-educational material on this process is recommended, in addition to resources that participants can carry with them to support this learning both during sessions and outside of them. | Mindfulness teacher incorporated this direction throughout the course. |
| 1.3. Adding elements  What trauma-specific elements should we add? | Agreement.  Trauma-specific psychoeducation [53, 55]. | NA | Agreement. Psychoeducational material on PTSD should be included in week 4 and delivered through experiential learning?  Yes (n=6)  Suggestions: written handouts, use Mindfulness Based Stress Reduction material on stress reactivity. | NA | Mindfulness teacher added trauma-specific psychoeducational component. |
| 1.4. Loosening structure: home practice  How can we support home practice? | No evidence on dose of home practice. | Agreement.  Women and professionals discussed: (i) the importance of practice outside of classroom setting; (ii) motivation to practice and prioritisation within busy life context; (iii) challenges to motivation if exercises feel irrelevant or negative in emphasis; (iv) recommendations for short, accessible practices, using different media; (v) the importance of media using female voices; (vi) safety concerns re home practice, if abuse is ongoing or level of trauma symptoms is high ; (vii) the importance of professionals holding non-judgemental attitudes and expectations about home practicing, for this to be discussed on a case-by-case basis at orientation | NA | NA | We developed a prototype coMforT app. |
| 1.5. Group size  What is the optimal group size? | Limited evidence.  4 - 8 participants in each group [55]. | NA | Agreement.  Minimum mean 6 (range 4-8)  Maximum mean 11 (range 8-25) | NA | Minimum 5, maximum 8 participants in each group. |
| 2. Course curriculum context (adaptations made to the way the course is delivered – format, setting, personnel, population) | | | | | |
| 2.1. Population: inclusion criteria  Should women be fluent in English? | Limited evidence.  Include those who understand spoken and written English [53]. | Agreement.  Women and professionals do not want to exclude people unnecessarily. Basic level English was considered a sufficient requirement and adaptions to materials, assessment at orientation. Since mindfulness is largely experiential, a future goal would involve delivery in different languages, | Agreement.  To include those who can understand and speak English but may not read or write (n=7). Suggestions: voice recording, idiosyncratic diaries, self-select at orientation, handouts in first language, course in first language. | NA | Inclusion criteria: can speak and understand English. |
| 2.2. Population: inclusion criteria  Should we include women with any PTSD and experience of DVA? | Agreement.  Include clinical PTSD, lifetime history of intimate partner violence [56].  Include varied levels of PTSD symptoms [19]. | Agreement.  Professionals felt that: (i) the assessment should screen for PTSD to avoid retraumatisation and triggering which could harm individuals/the group; (ii) people still processing trauma should not be offered mindfulness. Women described an escalation in PTSD symptoms with other therapies, and concern that this might happen with mindfulness. | NA | NA | Inclusion criteria: positive PTSD screen, service user at a specialist DVA agency. |
| 2.3. Population: exclusion criteria  What is the threshold for excluding women with current substance dependence? | Agreement.  Exclude current/recent substance abuse [53, 55, 56]. | Disagreement.  Women and professionals were undecided regarding substance dependence, which on one hand is a common coping mechanism, but may create barriers to engagement and impact on the group experience. Separate support may be needed. | Disagreement.  To invite women with current substance dependence to the orientation session where the teacher will assess each woman’s relationship to substance misuse and readiness to participate in an 8-week course?  Agree (n=6)  Disagree (n=2)  Exclusion can be a motivator to pursue sobriety. A shared investigation is needed, not the ‘teacher to assess’ as this mirrors ‘power-over’ mode of relationship. | Terms and conditions.  “not all substance dependence is equal”  There should be a manageable level of misuse (i.e., sober during each session). The teacher should use professional judgement to evaluate the level at individual orientation session: | Exclusion criteria: women who scored 20 and above on AUDIT (warrant further diagnostic evaluation for alcohol dependence)  and those scored 25 and above on DUDIT (probably heavily dependent on drugs).  At orientation session, the teacher will dialogue with the woman about their relationship to substance misuse and readiness to participate in an 8-week course. |
| 2.4. Population: exclusion criteria  Should we exclude women with self-harm and suicidal ideation? | No evidence. | Disagreement.  Women and professionals were undecided regarding self-harm and suicidal ideation, which on one hand are common in this population, but can be monitored, and additional support offered. Some professionals felt that people at high risk of suicide should be excluded. | Disagreement.  To invite women with suicidal ideations to the orientation session where the teacher will use professional judgement to assess woman’s readiness to participate in an 8-week course?  Agree (n=6)  Undecided (n=1)  Disagree (n=2) | Terms and conditions.  Take suicide history at baseline. Exclude those with: (i) suicide attempt in the past year, (ii) undertaken actions, (iii) having exact plans for suicide. Follow up everyone re: suicidal ideations. Have safety protocol in case of re-emerging suicidal ideation. | Exclusion criteria: suicidal attempt in the past year. |
| 2.5. Population: exclusion criteria  Should we exclude women who are currently experiencing DVA? | Agreement.  Exclude current IPV [53, 55, 56]. | Disagreement.  Women and professionals agreed that the decision of each individual was important re readiness and sustainability of separation. However, women highlighted issues re ongoing safety issues (court contact and stalking). And professionals highlighted difficulties re the cycle of abuse and coercive control. A few women felt that therapy should be available immediately after separation. | NA | NA | Included criteria: women who have already received services and risk assessment in a specialist DVA services. |
| 2.6. Population: exclusion criteria  Should we exclude women who are currently receiving any psychological treatment? | Disagreement.  The trauma-informed MBSR is not meant to be a standalone therapy for survivors of interpersonal trauma [53].  All participants were required to be in concurrent psychotherapy with a licensed practitioner [54].  Half of the studies used samples that had ongoing psychotherapy input [19]. | Disagreement.  Some professionals and women felt that therapies shouldn't run simultaneously due to potentially contradictory advice and practical issues. Others felt that therapies run in parallel would enable more effective support; with holistic approaches (mindfulness) and trauma-history therapies complimenting each other. | NA | NA | Exclusion criteria: current psychological therapy. |
| 2.7. Population: exclusion criteria  How should we evaluate individual woman’s readiness for engaging with mindfulness? | No evidence. | Agreement.  Professionals and women felt that psychological readiness for mindfulness was important, but that the stage and timeframe was different for individuals. A need for women to be open, and at a stage where they could manage the impact of therapy was important, and several women also highlighted the significance of recognising abuse as abuse – a process which continues through therapy. Professionals felt strongly that reflection is a key indicator of readiness, to be assessed at orientation. | NA | NA | Inclusion criteria: self-assessed psychological readiness for a psychological therapy. |
| 2.8. Population:  Can TS-MBCT be a first line therapy for PTSD in the DVA population? | Limited evidence.  Trauma-informed MBSR as a first-line therapy [53]. | Disagreement.  Most professionals felt that mindfulness should be offered after trauma-processing therapies and that a break between therapies is beneficial. A few felt that mindfulness could be a useful early intervention/part of a holistic approach. One woman highlighted the symptom focus of mindfulness (rather than an underlying trauma focus) and felt that being in a place of emotional stability was key. | Disagreement.  Trauma-informed MBCT is suitable as a first line therapy for DVA survivors with PTSD?  Yes (n=4)  Undecided (n=3)  No (n=1).  TS-MBCT with unprocessed trauma may increase PTSD symptoms so access to support is needed-may be a therapeutic opportunity rather than set back. | Terms and conditions.  Having received psychological therapy does not mean that the woman’s trauma has been processed. The individual’s readiness to process her trauma needs to be assessed at the orientation session. Safety policies and procedures should be in place that the woman knows who (e.g., general practitioner, DVA support worker, any mental health professional) and how to contact in case of adverse event. | Inclusion criteria: women exiting specialist DVA agency. This means that they received at least DVA advocacy.  Discuss support at orientation. Establish neutral foci of attention.  Provide guidance on how to work if triggered during TS-MBCT practices. |
| 2.9. Personnel: TS-MBCT teacher  What qualifications and experience should the teacher have? | Disagreement.  A licensed clinical social worker [53].  Qualified MBSR instructors [56, 57]. | Agreement.  Both women and professionals emphasised the need for facilitators to have expertise in trauma and DVA, alongside professional training in mindfulness. Women described the importance of facilitation skills which created a safe space; dealing with any arising trauma symptoms and difficult behaviours within the group.. Professionals felt that a minimum of two staff was necessary, and that facilitator lived experience was important for reducing power dynamics. . | Disagreement.  Teachers and teaching assistants delivering MBCT to trauma survivors should be registered psychotherapists and be trained in TS-MBCT?  Agree (n=3)  Undecided (n=1)  Disagree (n=3)  Essential qualities: strong, regular practice in compassion and equanimity and 1^st^ hand experience of working with trauma-but not necessarily as a psychotherapist- as there are many therapists who do not understand trauma. | Terms and conditions. Training in psychotherapy is desirable (helps to diagnose mental health comorbidities and manage risk) but not essential. Teachers with other professional mental health trainings (e.g., psychologists, nurses) should not be excluded from teaching TS-MBCT. The teacher and teaching assistant should have expertise in the population of trauma survivors. Personal experience of trauma can be counted towards such expertise. High prevalence of trauma experiences among general population supports the case for incorporating training on trauma (min 20 hours, webinar format) in the core training programme for MBSR/MBCT teachers. This is already happening in the US. Teachers delivering MBSR/MBCT to DVA survivors should receive additional training in trauma-specific mindfulness approaches. |  |
| 2.10. Participant-teacher and participant-participant relationships and dynamics  How should we manage group relationships and dynamics? | Limited evidence.  Some participants found the mindfulness group difficult at the beginning due to interpersonal dynamics with group members and becoming aware of their intense inner turmoil [49]. | Disagreement.  Both women and professionals described the benefits of groups for normalising experiences, and increasing feelings of safety. DVA awareness and active management of group dynamics by the facilitator were highlighted. Women felt that a supportive environment was created by being with other women who had had similar experiences. Professionals and women highlighted the challenges of building trust post-DVA in therapeutic relationships They described the importance of professionals being client-led, non-judgemental, and not manipulative to create a safe environment with balanced power relations. | Agreement.  What needs to be included in training to prevent a power imbalance in the teacher-participant relationship (free text)?  Asking permission, always offering choice; encouraging participants to honour their own truth and limits; explicitly addressing and working with that power differential as part of working through.  Include service users in the development, design and teaching of TS-MBCT.  Transference and counter transference. | Terms and conditions.  The learning environment in a mindfulness course is one of co-creation, in which the teacher and the participants are alongside each other in the process, rather than in a hierarchical relationship. Good mindfulness teaching involves invitations, rather than directions and promotes a sense of choice. In TS-MBCT, this feature is enhanced and made even more explicit: invitations, options and choices are constantly repeated, with participants being actively encouraged to see themselves as the experts in knowing what is best for them. Suggestions for further adaptations were made, which include some of the choices and safety mechanisms included in the Mindful Self-Compassion curriculum. Recommendations were offered for ways in which information about adverse effects of the course could be gathered from participants in a safe way. TS-MBCT teachers must have training in awareness of power differentials arising from class, race, education etc. |  |
| 2.11. Improving access to TS-MBCT or control interventions (access: timing, practical arrangements and setting)  How can we improve intervention accessibility? | Agreement.  Community-based settings [51, 53, 55, 56].  Reimbursed transport expenses [56].  Having their own transportation [55].  Providing childcare [56].  Pre-session group meals [56].  Weekly calls and reminders to attend the group [56]. | Agreement.  Both women and professionals highlighted the management of practical issues. Specifically, the need for flexibility around childcare (with a creche being ideal). Additionally, in the orientation, an assessment of housing, ongoing contact with perpetrator, and access to quiet, safe spaces for home practice is important.  Both women and professionals described the importance of therapist access outside of sessions. Professionals felt that this could be offered flexibly, and potentially over the phone. One woman suggested signposting information with contact details of other services. Others suggested a follow-up session after a period of time, to both reinforce learning and to assess longer term benefits. | NA | NA | TS-MBCT groups took place in a community centre accessible by public transport. We pre-booked taxies for women who preferred this transport. We reimbursed transport expenses and childcare. Therapist was available after each session and in-between via email/phone. |

Note. DVA, domestic violence and abuse. PTSD, post-traumatic stress disorder. MBSR, mindfulness-based stress reduction. MBCT, mindfulness-based cognitive therapy. TS-MBCT, trauma-specific mindfulness-based cognitive therapy. AUDIT, Alcohol Use Disorders Identification Test. DUDIT, Drug Use Disorders Identification Test.
