# Supplementary material for: Trauma-specific mindfulness-based cognitive therapy for women with post-traumatic stress disorder and a history of domestic abuse: intervention refinement and a randomised feasibility trial (coMforT study)
Source: Pilot Feasibility Stud. 2023 Jul 3;9:112. doi: 10.1186/s40814-023-01335-w (PMC10316568; doi:10.1186/s40814-023-01335-w)
Supplement: Supplementary file 5 — Additional file 5: Supplementary file 5. Raw candidate clinical outcomes at baseline and 6-month follow-up. [file 40814_2023_1335_MOESM5_ESM.docx]

Supplementary file 5. Candidate clinical outcomes at baseline and 6-month follow-up stratified by trial arm

|  | **Baseline** | | | | | | **6-month follow-up** | | | | |
| --- | --- | --- | --- | --- | --- | --- | --- | --- | --- | --- | --- |
|  | **Intervention (n=15)** | | | **Control (n=5)** | | | **Intervention (n=13)** | | | **Control (n=3)** | |
|  | **N** | **Mean (95% CI) or % (95% CI) if stated** | **N** | | **Mean (95% CI) or % (95% CI) if stated** | **N** | | **Mean (95% CI) or % (95% CI) if stated** | **N** | | **Mean (95% CI) or % (95% CI) if stated** |
| **Primary outcomes** | | | | | | | | | | | |
| Clinically important symptoms of PTSD, PTSD Checklist DSM-5 | 14 | 49.1 (41.0, 57.2) | 5 | | 61.0 (46.7, 75.3) | 12 | | 30.8 (19.3, 42.2) | 3 | | 47.3 (-4.4, 99.0) |
| Clinically important symptoms of PTSD, PTSD Checklist DSM-5 diagnosis, n(%) | 15 | 86.7% (59.5%, 98.3%) | 5 | | 100% (47.8%, 100%) | 13 | | 61.5% (31.6%, 86.1%) | 3 | | 66.7% (9.4%, 99.2%) |
| Clinically important symptoms of PTSD, International Trauma Questionnaire – PTSD score | 15 | 15.3 (12.3, 18.4) | 5 | | 19.4 (13.8, 25.0) | 13 | | 8.5 (4.4, 12.6) | 3 | | 14.0 (5.0, 23.0) |
| International Trauma Questionnaire – DSO score | 14 | 16.1 (12.6, 19.7) | 5 | | 17.8 (11.5, 24.1) | 12 | | 9.9 (5.7, 14.1) | 3 | | 17.3 (1.4, 33.3) |
| Clinically important symptoms of PTSD and CPTSD, International Trauma Questionnaire diagnoses n(%) : |  |  |  | |  |  | |  |  | |  |
| - PTSD | 15 | 6.7% (0%, 29.7%) | 5 | | 20% (0%, 74.1%) | 13 | | 7.7% (0%, 31.9%) | 3 | | 0% (0%, 56.4%) |
| - CPTSD | 15 | 66.7% (46.7%, 89.7%) | 5 | | 60% (40.0%, 100%) | 13 | | 15.4% (0%, 39.6%) | 3 | | 0% (0%, 56.4%) |
| **Secondary outcomes** | | | | | | | | | | | |
| Dissociative symptoms, DES-B | 15 | 9.4 (6.7, 12.1) | 5 | | 9.2 (4.3, 14.1) | 13 | | 7.8 (4.6, 11.0) | 3 | | 11.3 (-5.2, 27.9) |
| Depression, PHQ-9 | 15 | 14.4 (11.1, 17.7) | 5 | | 17.8 (8.0, 27.6) | 13 | | 9.2 (6.1, 12.3) | 2 | | 21.5 (-10.3, 53.3) |
| Anxiety, GAD-7 | 14 | 13.4 (10.5, 16.3) | 5 | | 18.2 (15.2, 21.2) | 12 | | 6.6 (4.2, 9.0) | 2 | | 12.5 (-57.4, 82.4) |
| Self-compassion | 15 | 2.7 (2.3, 3.0) | 5 | | 1.9 (1.5, 2.4) | 13 | | 3.0 (2.6, 3.5) | 3 | | 2.3 (0.8, 3.7) |
| Intimate partner violence score in past 6 months | 13 | 13.0 (5.5, 20.5) | 5 | | 32.9 (13.9, 51.9) | 12 | | 3.6 (1.0, 6.2) | 2 | | 10.5 (-8.6, 29.6) |

Note. N, completed questionnaire. CI, confidence interval. Confidence intervals for proportions have been calculated using the Clopper-Pearson binomial method. Confidence intervals for multinomial proportions (from ITQ) have been calculated using the Glaz and Sison method. PTSD, post-traumatic stress disorder. CPTSD, complex post-traumatic stress disorder. DSM-5, Diagnostic and Statistical Manual of Mental Disorders, 5th Edition. PHQ-9, Patient Health Questionnaire. GAD-7, Generalised Anxiety Disorder questionnaire.
